# Genome-wide diversity and differentiation in New World populations of the human malaria parasite *Plasmodium vivax*

**DOI:** 10.1371/journal.pntd.0005824

**Published:** 2017-07-31

**Authors:** Thais C. de Oliveira, Priscila T. Rodrigues, Maria José Menezes, Raquel M. Gonçalves-Lopes, Melissa S. Bastos, Nathália F. Lima, Susana Barbosa, Alexandra L. Gerber, Guilherme Loss de Morais, Luisa Berná, Jody Phelan, Carlos Robello, Ana Tereza R. de Vasconcelos, João Marcelo P. Alves, Marcelo U. Ferreira

**Affiliations:** 1 Department of Parasitology, Institute of Biomedical Sciences, University of São Paulo, São Paulo, Brazil; 2 Unit of Computational Genomics Darcy Fontoura de Almeida, Laboratory of Bioinformatics, National Laboratory of Scientific Computation, Petrópolis, Brazil; 3 Unit of Molecular Biology, Pasteur Institute of Montevideo, Montevideo, Uruguay; 4 London School of Hygiene and Tropical Medicine, London, United Kingdom; Johns Hopkins Bloomberg School of Public Health, UNITED STATES

## Abstract

**Background:**

The Americas were the last continent colonized by humans carrying malaria parasites. *Plasmodium falciparum* from the New World shows very little genetic diversity and greater linkage disequilibrium, compared with its African counterparts, and is clearly subdivided into local, highly divergent populations. However, limited available data have revealed extensive genetic diversity in American populations of another major human malaria parasite, *P*. *vivax*.

**Methods:**

We used an improved sample preparation strategy and next-generation sequencing to characterize 9 high-quality *P*. *vivax* genome sequences from northwestern Brazil. These new data were compared with publicly available sequences from recently sampled clinical *P*. *vivax* isolates from Brazil (BRA, total n = 11 sequences), Peru (PER, n = 23), Colombia (COL, n = 31), and Mexico (MEX, n = 19).

**Principal findings/Conclusions:**

We found that New World populations of *P*. *vivax* are as diverse (nucleotide diversity π between 5.2 × 10^−4^ and 6.2 × 10^−4^) as *P*. *vivax* populations from Southeast Asia, where malaria transmission is substantially more intense. They display several non-synonymous nucleotide substitutions (some of them previously undescribed) in genes known or suspected to be involved in antimalarial drug resistance, such as *dhfr*, *dhps*, *mdr1*, *mrp1*, and *mrp-2*, but not in the chloroquine resistance transporter ortholog (*crt-o*) gene. Moreover, *P*. *vivax* in the Americas is much less geographically substructured than local *P*. *falciparum* populations, with relatively little between-population genome-wide differentiation (pairwise *F*_ST_ values ranging between 0.025 and 0.092). Finally, *P*. *vivax* populations show a rapid decline in linkage disequilibrium with increasing distance between pairs of polymorphic sites, consistent with very frequent outcrossing. We hypothesize that the high diversity of present-day *P*. *vivax* lineages in the Americas originated from successive migratory waves and subsequent admixture between parasite lineages from geographically diverse sites. Further genome-wide analyses are required to test the demographic scenario suggested by our data.

## Author summary

*Plasmodium vivax* is the most common human malaria parasite in the Americas, but how and when this species arrived in the New World remains unclear. Here we describe high-quality whole-genome sequence data for nine *P*. *vivax* isolates from Brazil, a country that accounts for 37% of the malaria burden in this continent, and compare these data with additional publicly available *P*. *vivax* genomes from Brazil, Peru, Colombia, and Mexico. *P*. *vivax* populations from the New World were found to be as diverse as their counterparts from areas with substantially higher malaria transmission, such as Southeast Asia, and to carry several non-synonymous substitutions in candidate drug-resistance genes. Moreover, genome-wide patterns of linkage disequilibrium between pairs of polymorphic sites are consistent with very frequent outcrossing in these populations. Interestingly, local *P*. *vivax* is more polymorphic, with less between-population differentiation, than sympatric populations of *P*. *falciparum*, possibly as a result of different demographic histories of these two species in the Americas. We hypothesize that local *P*. *vivax* lineages originated from successive migratory waves and subsequent admixture between parasites from geographically diverse sites.

## Introduction

*Plasmodium vivax* is the human malaria parasite with the widest global distribution and accounts for nearly half of the combined malaria burden in South and Southeast Asia, Oceania, and Central and South America. Over one-third of the world's population is currently at risk of infection with this species, with 16 million clinical cases recorded each year [1]. Although *P*. *vivax* has most likely evolved from parasites that infect chimpanzees and gorillas in sub-Saharan Africa [2,3], it is nowadays rare in most of this continent, where human populations lack a key erythrocyte receptor for host cell invasion by blood-stage parasites, the Duffy antigen/receptor for chemokines (DARC) [4]. Where both species coexist, *P*. *vivax* typically causes less severe cases and fewer deaths than *P*. *falciparum*, the most virulent human malaria parasite, but represents a major challenge for ongoing malaria elimination efforts worldwide [1].

The Americas were the last continent colonized by humans carrying malaria parasites, but the dates and routes of migration of *P*. *vivax* to the New World are still debated [5–7]. Archaeological evidence for infection with this parasite in indigenous, pre-Columbian populations is currently limited to a single report of *P*. *vivax* antigens being visualized by immunohistochemistry in the liver and spleen of South American mummies dating from 3,000 to 600 years ago [8]. Interestingly, specific antibodies failed to detect *P*. *falciparum* antigens in these same samples [8]. These findings are consistent with the hypothesis that *P*. *vivax*, but not *P*. *falciparum*, was brought to the New World by early human migrations from East Asia or the Western Pacific [5], but more specific molecular techniques are required to confirm them [9]. Nevertheless, present-day New World populations of *P*. *vivax* appear to be more closely related to extant African and South Asian parasites and now extinct European lineages than to East Asian and Melanesian strains [10–12], consistent with much more recent parasite migrations with European conquerors and African slaves during the colonial era [7,13].

Clinical isolates from Brazil are underrepresented in global genomic analyses of *P*. *vivax* [11,14]. However, this country contributes 37% of the malaria burden in the Americas, a region with over 20 million people at high risk of infection [1]. Obtaining large amounts of host-free *P*. *vivax* DNA from clinical samples from Brazil has been a major challenge for genome sequencing projects, because (a) blood-stage parasite densities are typically very low [15], (b) clinical blood samples are heavily contaminated with human DNA from leukocytes, and (c) methods for long-term *in vitro* propagation of *P*. *vivax* are neither practical nor widely reproducible [16–18]. Here, we combined an improved sample preparation strategy, for reducing human DNA contamination and increasing target parasite's DNA yield, with next-generation genome re-sequencing to examine a local population of *P*. *vivax* from the Amazon Basin of Brazil. Our nine high-quality genome sequences were compared to those previously obtained from four countries (Brazil [BRA], Peru [PER], Colombia [COL], and Mexico [MEX]) [11,14] to reveal local and regional patterns of diversity and differentiation in extant *P*. *vivax* populations from the New World.

## Methods

### Ethics statement

Study protocols were approved by the Institutional Review Board of the Institute of Biomedical Sciences, University of São Paulo, Brazil (936/CEP, 2010 and 1183/CEPSH, 2014). Written informed consent was obtained from all patients.

### Sample collection

**Fig 1:**
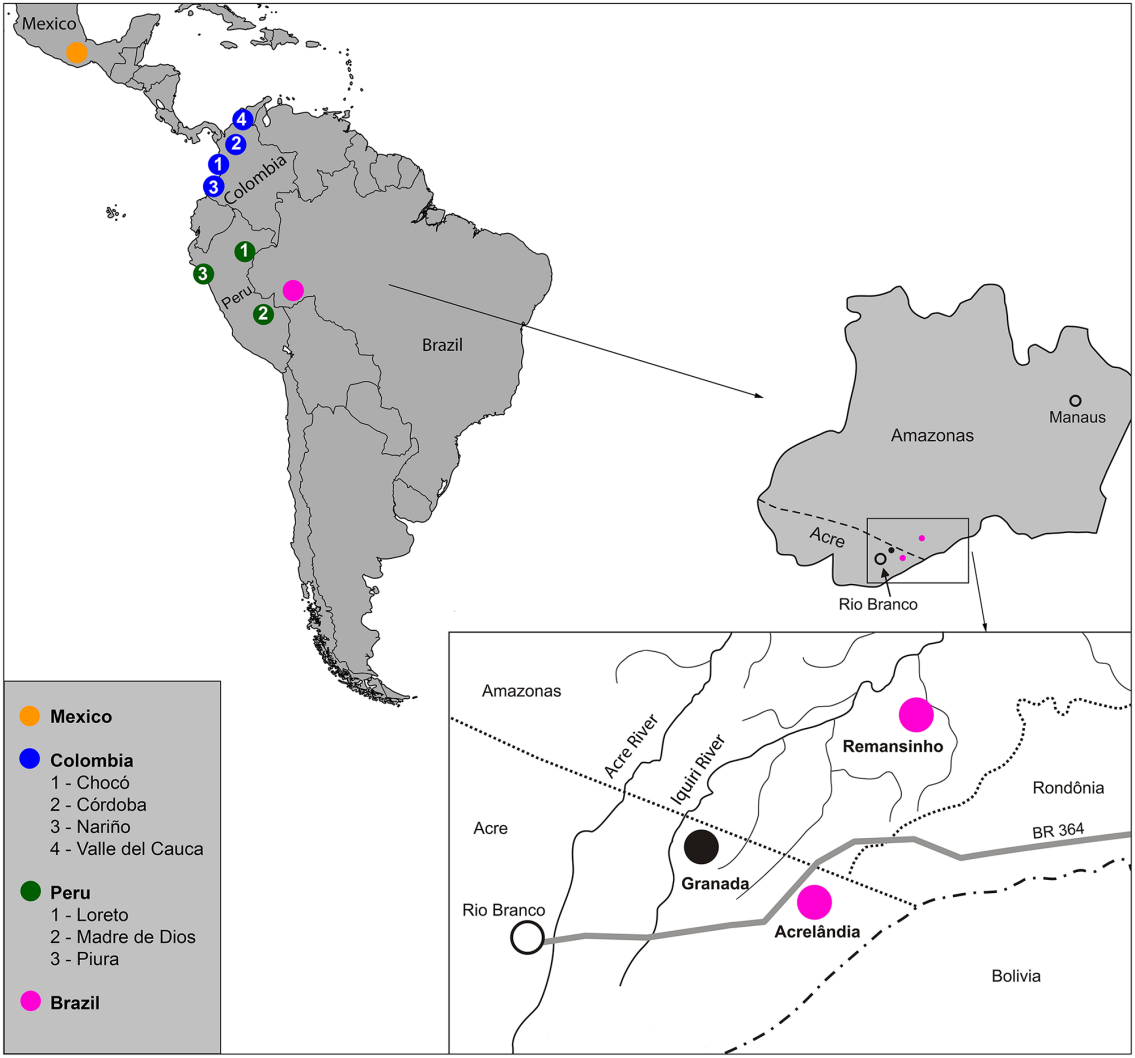
Map showing the sampling locations of the New World *P*. *vivax* isolates analyzed in this study, comprising Brazil, Peru, Colombia, and Mexico. The insert shows the study sites in northwestern Brazil (Acrelândia and Remansinho, 120 km apart), close to the border with Bolivia and Peru. Adapted from [15] and https://commons.wikimedia.org/wiki/Atlas_of_the_world#/media/File:BlankMap-World6.svg.

Parasite samples were collected between November 2012 and June 2013 in eastern Acre and southern Amazonas, Amazon Basin of Brazil, close to the borders with Peru and Bolivia. Malaria epidemiology in the study sites has been characterized in detail elsewhere [15,19]. Venous blood samples (10 ml) were collected from eight adult patients attending malaria clinics in the town of Acrelândia, Acre (9°43' S, 66°53' W), and one adult patient living in the farming settlement of Remansinho, Amazonas (9°40' S-9°43' S, 66°52' W -66°59' W), 120 km east of Acrelândia (Fig 1). *P*. *vivax* infection was diagnosed by on-site microscopy and later confirmed by qPCR as described below.

### Leukocyte depletion and sample cryopreservation

We adapted BioR 01 Plus leukocyte-depletion filters (Fresenius Kabi, Bad Homburg, Germany; S1A Fig) to process 10 to 50-ml volumes of venous blood in our field laboratory in the Amazon [20]. The per-unit cost of these filters in Brazil is around US$ 25. We first cut off under sterile conditions, with a scissor, the tubing that connects the filtering device to the 400-ml blood storage bag and to the adapter (S1B and S1C Fig). S1D Fig shows how the filtering device was used in a laminar flow hood; briefly, a 10-ml syringe was used to apply blood treated with acid citrate dextrose anticoagulant, while a second 10-ml syringe was adapted to the end of the remaining tubing to recover the filtered, leucocyte-depleted material, which was transferred to 50-ml sterile centrifuge tubes. No priming with saline was required. After the filtration process, the leukocyte depletion device was washed through with at least twice the volume of RPMI medium as the original blood sample to recover red blood cells (RBCs) that had been retained in the filter and tubing. Next, the mixture of filtered blood and RPMI medium recovered in 50-ml tubes was centrifuged at 800 *g* for 5 minutes and the supernatant (plasma plus RPMI medium) was removed with a sterile Pasteur pipette.

For cryopreservation, the RBC pellet was resuspended dropwise in Glycerolyte-57 solution (Fenwall, Fresenius Kabi), at the proportion of 1.66 ml of Glycerolyte for each 1 ml of cell pellet, under gentle agitation. One ml aliquots of the RBC-Glycerolyte mixture were transferred to screw-capped cryovials and placed in a Nalgene Mr. Frosty freezing container (ThermoFisher Scientific, Waltham, MA) that was kept at -80°C for 24 hours. The next day, cryovials were plunged in liquid nitrogen for long-term storage. Samples were shipped to São Paulo in liquid nitrogen, for subsequent schizont maturation.

### Short-term in vitro culture for *P*. *vivax* schizont maturation and chloroquine resistance testing

For sample thawing, cryovials were removed from the liquid nitrogen and maintained for 1 min at room temperature (20–25°C) and 1 min at 37°C. The 1-ml samples were then transferred to 50-ml centrifuge tubes, gently mixed with 200 μl of 12% NaCl solution, and let stand for 1 minute. Next, 10 ml of 1.6% NaCl were gently added, the mixture was gently agitated, and centrifuged at 180 *g* for 8 min [21]. After removing the supernatant, the RBC pellet was washed twice in incomplete McCoy's 5A medium supplemented with glucose (0.5% w/v), HEPES (25 mM), and hypoxantine (0.005% w/v), and resuspended in complete McCoy medium (as above but supplemented with 25% AB+ heat-inactivated human serum) to a final hematocrit of 2%. Short-term culture in vitro was carried in flat-bottomed dishes placed in a gas chamber with controlled O_2_ and CO_2_ levels that was kept at 37°C for up to 48 hours. Parasite growth and maturation were monitored as described [17].

Chloroquine resistance (CQR) was evaluated using an *ex-vivo* schizont maturation assay in selected *P*. *vivax* samples with > 1,000 parasites/μl of blood and > 50% ring stages at the time of thawing [22]. There is no consensus regarding the 50% inhibitory concentration (IC_50_) indicative of CQR in *P*. *vivax*; suggested cut off values range between 100 nM [23] and 220 nM [24], but all IC_50_ values in our samples were < 50 nM (Table 1).

**Table 1:** Field-collected *Plasmodium vivax* isolates from Brazil with nuclear genomes newly sequenced in this study and summary of sequencing statistics.

Isolate	Site^a^	Date of collection	IC_50_ for CQ^b^(mM)	No. reads (× 10^6^)	Percent reads mapped to Sal-I^c^	Average sequence depth	Percent Sal-I genome covered	No. SNPs^d^
**17**	Acrelândia	Jun/13	11.3	17.85	95%	49.79×	84%	6445
**18**	Acrelândia	Dec/12	--^e^	23.40	93%	59.35×	85%	6232
**19**	Acrelândia	Nov/12	11.1	23.87	95%	71.93×	86%	5524
**20**	Acrelândia	Apr/13	14.5	18.80	93%	58.91×	89%	6188
**32**	Acrelândia	Jun/13	20.5	22.51	92%	69.09×	93%	5528
**51**	Acrelândia	Jun/13	--	20.93	94%	58.24×	83%	5819
**52**	Acrelândia	Jun/13	--	21.43	94%	62.90×	89%	6158
**207**	Remansinho	Apr/13	46.7	21.08	95%	70.13×	89%	6041
**ACR**	Acrelândia	Nov/12	20.9	21.04	93%	78.65×	92%	5414
**Average**				21.21	94%	64.33×	88%	5927.7

^a^Sample collection sites are shown in Fig 1.

^b^50% inhibitory concentration for chloroquine (CQ) determined by ex-vivo schizont maturation test.

^c^Sal I = reference assembled genome, *P*. *vivax* monkey-adapted strain Sal 1 [61]

^d^Number of remaining high-quality single-nucleotide polymorphisms (SNPs) after applying the quality filters described in the main text.

^e^--not determined.

### DNA isolation and quantification of human and *P*. *vivax* DNA with real-time PCR

DNA templates were isolated from 200-μl aliquots of either whole venous blood (before leukocyte removal) or RBC pellet (after leukocyte removal) using QIAamp DNA blood kits (Qiagen, Hilden, Germany). To estimate the relative proportion of human and parasite DNA, we used SYBR Green qPCR targeting single-copy genes coding for human topoisomerase III and *P*. *vivax* aldolase. Each 15 μl qPCR mixture contained 2 μl of template DNA, 7.5 μl of 2× Maxima SYBR Green qPCR master mixture (Fermentas, Burlington, Canada) and 0.3 μM of each of the primer pairs, *Pvaldo*-F (GAC AGT GCC ACC ATC CTT ACC) plus *Pvaldo*-R (CCT TCT CAA CAT TCT CCT TCT TTC C) and *Top3*-F (CAT GTT TGA GCT GAG CCT GA) plus *Top3*-R (CCA CAC CAC ACC CCT AAC TT). Standard curves were prepared with serial tenfold dilutions of a plasmid containing both target sequences to allow for copy number quantitation (number of amplicons/μl of blood). We used a Step One Plus Real-Time PCR System (Applied Biosystems, Foster City, CA) for PCR amplification with a template denaturation step at 95°C for 10 min, followed by 40 cycles of 15 sec at 95°C and 1 minute at 60°C, with fluorescence acquisition at the end of each extension step. Amplification was followed by a melting program consisting of 15 sec at 95°C, 15 sec at 60°C, and a stepwise temperature increase of 0.2°C/sec until 95°C, with fluorescence acquisition at each temperature transition. All reactions were made in triplicate. We measured parasite DNA enrichment as the parasite:human copy-number ratio after filtering divided by parasite:human copy-number ratio before filtering. To estimate the proportion of host and parasite DNA in each sample, we considered the amplicon copy numbers and the genome size of humans (3.2 Gb) and *P*. *vivax* (26.8 Mb; Sal-I assembly); the DNA content in each copy of the human genome corresponds approximately to that of 119 copies of the *P*. *vivax* genome.

### DNA library preparation and whole-genome sequencing

Parasite DNA templates were quantified by fluorometry using a Qubit 3.0 fluorometer (Invitrogen, Carlsbad, CA) and sequenced using Ion Torrent Personal Genome Machine (PGM) and Ion Proton platforms (Life Technologies, Foster City, CA) at the Unit of Computational Genomics, Laboratory of Bioinformatics, National Laboratory of Scientific Computation, Brazil. Separate libraries were prepared for each sequencing platform, using 1 μg of template DNA per isolate. DNA samples were sheared using the Bioruptor UCD-200 TS (Diagenode, Liege, Belgium) sonication system until fragment sizes of 200 bp (for Ion PGM libraries) or 150 bp (for Ion Proton libraries) were obtained. Libraries were prepared using the Ion Xpress Plus Fragment Library kit, with Ion Xpress Barcode adapters according to the Ion Xpress Plus gDNA Fragment Library Preparation protocol (Life Technologies). Size selection was performed on E-Gel SizeSelect 2% agarose gels using the E-Gel iBase Power System (ThermoFisher Scientific). Emulsion PCR was done on the Ion OneTouch 2 system (Life Technologies) with the Ion PGM Template OT2 200 kit or the Ion PI Template OT2 200 kit version 2 for Ion PGM and Ion Proton, respectively, following the manufacturer's instructions (Life Technologies). Ion PGM libraries were loaded on Ion318 chips v2 and sequenced using the Ion PGM Sequencing 200 kit v2; Ion Proton libraries were loaded on Ion PI chips v2 and sequenced using the Ion PI Sequencing 200 kit v2 (Life Technologies). All samples were sequenced on both platforms; sequence reads (150–200 bp) from two runs in each platform were merged into a single fastq file per sample.

### Additional genomic data sets

To place our genomic data in a regional context, we reanalyzed raw paired-end Illumina reads from 107 additional *P*. *vivax* clinical isolates from the Americas. Fastq files were downloaded from the Sequence Read Archive (SRA) of the National Center for Biotechnology Information, United States, and processed in the same way as our newly obtained sequences. Three clinical isolates from Brazil had sequence data generated on an Illumina Genome Analyzer II platform at the Welcome Trust Sanger Institute (Hinxton, Cambridge, UK), as part of the *P*. *vivax* genome variation project coordinated by the MalariaGEN network [14]. Other isolates—20 from Brazil, 34 from Peru, 31 from Colombia, and 19 from Mexico—had whole-genome sequence data generated on an Illumina HiSeq 2000 platform at the Broad Institute of MIT and Harvard (Cambridge, MA, USA), as part of the International Centers of Excellence for Malaria Research (ICEMR) program [11]. All isolates from Brazil sequenced in these two previous studies were collected in endemic areas surrounding Acrelândia, Acre State, between 2008 and 2011.

### Single-nucleotide polymorphism (SNP) calling

Fastq files were first filtered for quality; 8 SRA samples were excluded from further analysis because of mean quality scores ≤ 30 (expected base call accuracy ≤ 99.9%). We next mapped the high quality reads onto the PlasmoDB version 10.0 of the Sal-1 reference (http://plasmodb.org/common/downloads/release-10.0/PvivaxSal1/fasta/data/PlasmoDB-10.0_PvivaxSal1_Genome.fasta) [25], using Bowtie2 version 2.2.6 [26] with the “very sensitive” preset, allowing one mismatch per seed region; 11 SRA samples were excluded at this stage because of < 60% mapping over the reference. The resulting alignments were merged into BAM files with SAMtools [27], duplicate reads were identified and marked using the Picard version 2.0.1 MarkDuplicates tool, and files were indexed with SAMtools.

We used GATK version 2.0 [28] for SNP calling following the GATK Best Practices (https://software.broadinstitute.org/gatk/best-practices/). GATK UnifiedGenotyper with the BaseAlignmentQuality option was used to obtain high-confidence SNPs by applying stringent VariantFiltration criteria: (a) coverage > 20×, (b) mapping quality > 30, (c) base quality > 30, read depth and allelic fraction by sample ≥ 1, and (d) haplotype score ≤ 3. We removed all SNPs with > 2 alleles and those with minor allele frequency < 0.01 (reads counted across all sequenced samples).

SnpEff [29] was used to identify SNPs mapping to coding sequences (further classified as synonymous or nonsynonymous), introns, and intergenic regions of the Sal-I reference genome. The resulting catalogue of 94,122 high-confidence SNPs was used to genotype each individual sample using GATK UnifiedGenotyper with default parameters except for the minimum phred-scaled confidence threshold, with a calling variant = 50 and emitting variant = 10. Heterozygote calls were converted to the majority allele if ≥75% of the reads in that sample were the majority allele; otherwise, the allele was undetermined. Sites with < 5× coverage in a given sample were filtered out at this stage.

The final data set of *P*. *vivax* nuclear genome sequences from the New World comprised our 9 newly sequenced samples from BRA and 75 high-quality SRA samples (2 from BRA, 23 from PER, 31 from COL, and 19 from MEX); 13 SRA samples were removed during the genotyping process because the number of SNPs identified (range: 13 to 2,889) was below the predefined minimum of 3,000 nucleotide differences compared to the Sal-I reference, consistent with poor sequencing coverage. Overall, we excluded 21 (out of 23) SRA samples from BRA and 11 (out of 34) SRA samples from PER at different stages of this analysis. Isolate codes and SRA accession numbers of samples used in this analysis are given in S1 Table.

### Within-population genetic diversity and recombination

The average pairwise nucleotide diversity (π, average number of nucleotide differences per site between pairs of DNA sequences) was calculated within each geographic population using VCFtools [30]. Values were plotted, using R version 3.3.0, as moving averages within 1-kb sliding windows across each chromosome. We recalculated π after masking out subtelomeric regions and three hypervariable internal chromosome regions (containing *sera*, *msp-3*, and *msp-7* gene families) that were more prone to sequence misalignments and poor read mapping in a previous analysis [14]. The coordinates of these regions are given in the Supplementary Table 2 of Pearson et al. [14].

Similarly, Tajima's *D* values [31] were calculated using VCFtools; mean Tajima’s *D* across 1-kb windows were plotted for each population. Frequency distributions of π and Tajima’s *D* values within 1-kb windows were plotted for each population. We defined windows with the top 50 π values within each population as highly variable genomic regions. We defined as outliers the 1-kb windows with the 50 highest and 50 lowest mean Tajima's *D* values within each population. We also examined the minor allele frequency (MAF) spectrum separately in each population.

We next estimated, for each population, the rate at which pairwise linkage disequilibrium (LD) decreased with increasing physical distance between SNPs due to meiotic recombination. The squared correlation coefficient *r*^2^ between pairs of SNPs of varying distance across the same chromosome was measured using VCFtools; *r*^2^ values were binned by distance (50-bp windows) and medians within each window were plotted against physical distance between SNPs. The level of background LD between unlinked markers within each population was estimated by calculating median *r*^2^ between all pairs of SNPs on different chromosomes.

### Between-population genetic differentiation and population structure

The Wright's fixation index *F*_ST_, a measure of population differentiation due to genetic structure [32], was calculated with VCFtools for each SNP in every pairwise comparison of populations. Values were averaged across all SNPs to estimate overall pairwise differentiation between populations. The 100 SNPs with the highest average *F*_ST_ values across all populations were further characterized.

To assess population structure, we first used the PLINK software (https://www.cog-genomics.org/plink2 [33]) to carry out principal component analysis (PCA); up to 10 components were analyzed. For phylogenetic analysis, a neighbor-joining tree was constructed via the maximum composite likelihood substitution model with 1,000 bootstrap pseudoreplicates using MEGA 7.0 (http://www.megasoftware.net/). To estimate the ancestry shared between individual isolates, we used the ADMIXTURE software package [34] with either all 94,122 high-quality SNPs or a curtailed set of 12,762 SNPs that are not linked. To this end, we removed each SNP that had an *r*^2^ value > 0.1 with any other SNP within a 60-SNP sliding window advanced by 10 SNPs each time. The optimal number of clusters (*K*) was determined by performing multiple runs of the software under different *K* values (2–10) and selecting the *K* values (*K* = 2 and *K* = 3) associated with the lowest cross-validation error compared to other *K* values (S2 Fig).

### Data availability

The sequence data supporting the conclusions of this article are available in the Sequence Read Archive of the National Center for Biotechnology Information, United States; accession numbers are provided in S1 Table.

## Results and discussion

### Sample preparation and genome sequencing

We designed a single-step procedure to reduce human DNA content in *P*. *vivax*-infected blood using commercially available leukocyte-depletion filters (S1 Fig). Leucocyte depletion in 17 clinical samples, with initial parasitemias ranging between 854 and 43,177 (median: 7,566) parasites/μl, decreased the percent human DNA content from a median of 99.2% (range: 82.7–99.9%) to 23.3% (range: 0–97.6%). No human DNA could be detected by quantitative real-time polymerase chain reaction (qPCR) in three filtered samples (i.e., 0% host DNA contamination). The residual human DNA content in our leukocyte-depleted samples was similar to the median of 33.9% (range: 1.6–68.6%) found in blood samples from Indonesia after double filtration through CF-11 cellulose columns [17]. The increase in parasite:human DNA ratio after leukocyte depletion ranged between 1.7 and 3,060-fold (median: 227-fold) and appeared to be inversely proportional to the initial, pre-treatment parasite:human DNA ratio (Spearman correlation coefficient *r*_s_ = -0.512, *P* = 0.061; S3 Fig), but did not correlate with the initial parasitemia (*r*_s_ = 0.165, *P* = 0.412).

The next challenge consisted in selectively increasing parasite DNA yield for genome sequencing. To this end, leukocyte-depleted *P*. *vivax* samples were cultured *in vitro* for up to 44 h to allow uninucleate trophozoites to mature to multinucleate blood-stage schizonts, increasing parasite DNA content by ≥4-fold [17]. We obtained enough template DNA for library preparation and sequencing from 9 cultured samples, achieving 49.8–78.6× average genome sequencing depth; 91.8–94.7% of the reads mapped to reference Sal-I genome (Table 1). These results compared favorably with the coverage and percentage of reads mapping onto the reference *P*. *vivax* genome obtained with other different methods for clinical sample preparation: (a) single CF-11 column filtration (0.8–42.1× depth and 0.4–27.8% mapping in 8 isolates from Colombia [35] and 4.2–28.1× depth and 18.3–55.4% mapping in 11 isolates from Peru [36,37]), (b) double CF-11 column filtration (70–407× depth and 15.0–46.2% mapping in 5 isolates from Cambodia and Madagascar [38]), (c) double CF-11 column filtration followed by in vitro schizont maturation (18–116× depth and 11.0–89.1% mapping in 22 isolates from Thailand and returning travelers [17]), and (d) in-situ hybridization for *P*. *vivax* whole-genome capture in unfiltered samples (21.9–160.4× depth and 20.2–80.1% mapping in 5 isolates from Peru [16] and 34.8–118.2× depth and 19.6–39.7% mapping in 3 isolates from East Asia [39]). Therefore, the sample preparation strategy described here allowed for high sequence coverage and depth, but further comparisons are limited by the use of different next-generation sequencing platforms across studies (Ion PGM and Ion Proton here and Illumina in other studies).

### Local and regional patterns of genome-wide diversity

We used the Ion PGM and Ion Proton platforms to generate between 17.8 and 23.9 million sequence reads from 9 clinical samples of *P*. *vivax* from Brazil (Table 1). To explore local levels of genomic diversity, our sequence data were combined with those from two *P*. *vivax* clinical samples from Brazil that were previously obtained with Illumina platforms [11,14]. Sequence reads from these additional samples (PV4 and Brazil32) covered 86.2% and 95.0% of the reference genome, respectively (S1 Table). Since all isolates from Brazil (n = 11) were collected from sites within a radius of 120 km in the Amazon Basin (Fig 1), we define BRA as a local, nearly sympatric *P*. *vivax* population. After applying stringent quality control filters to raw sequence reads (see Methods), we uncovered 27,360 high-confidence single-nucleotide polymorphisms (SNPs) in the BRA population. The overall nucleotide diversity π was estimated at 5.6 x 10^−4^).

**Fig 2:**
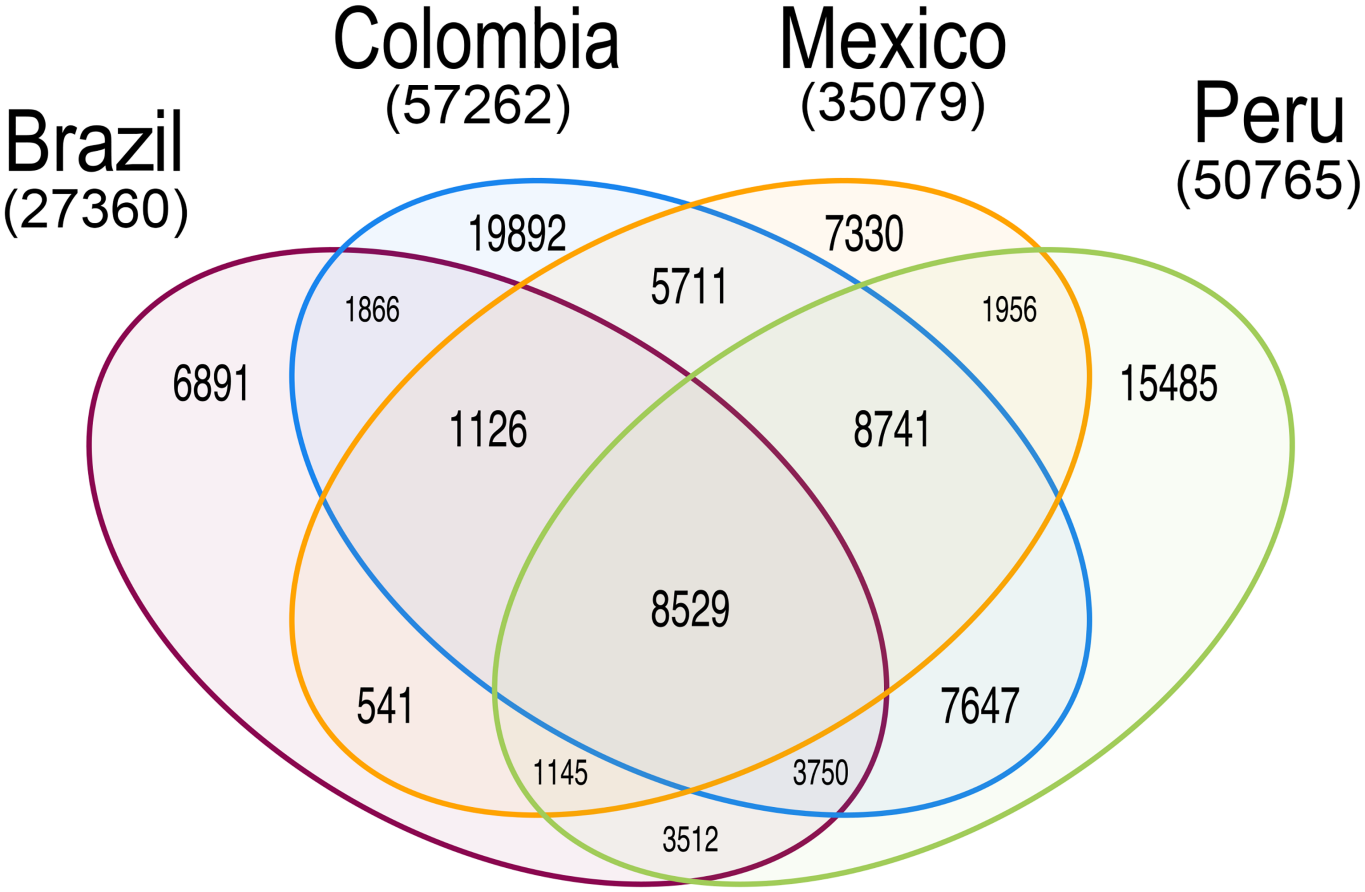
Venn diagram showing the number of SNPs shared by *P*. *vivax* samples from Brazil (n = 11 isolates), Peru (n = 23), Colombia (n = 31), and Mexico (n = 19).

We next compared BRA sequence data with those from three other New World populations of *P*. *vivax*: PER (n = 23), COL (n = 31), and MEX (n = 19) [11], from clinical isolates sampled in sites shown in Fig 1. Two populations were geographically heterogeneous: PER samples were collected in three departments (Loreto and Madre de Dios, both in the Amazon Basin, and Piura, on the northwestern Pacific coast), while COL samples came from four departments (Nariño, Valle del Cauca, and Chocó along the Pacific Coast, and Córdoba, on the Caribbean coast). MEX samples were from five different sites, but all in the southern state of Chiapas (S1 Table). We characterized 94,122 biallelic SNPs passing our high-quality filters in 84 samples; 55.0% of them are located in intergenic regions, 8.8% in introns, and 36.2% in coding regions. Most (61.2%) coding SNPs were non-synonymous (nsSNPs), as previously found in other regional *P*. *vivax* populations [14,38]. Unsurprisingly, the number of SNPs found in each population was directly proportional to sample size, being lowest in BRA (n = 27,360) and highest in COL (n = 57,262; Fig 2). Overall, 49,598 (52.3%) SNPs were unique to a population (i.e., private) and 8,529 (9.1%) were shared by all populations (Fig 2). Of the 6,891 private SNPs found in BRA, 40.0% mapped to coding sequences; 65.2% of the coding SNPs were non-synonymous. PER and COL shared the highest number of SNPs (n = 28,667), followed by COL and MEX (n = 24,107) (Fig 2).

Nucleotide diversity in PER (π = 5.2 x 10^−4^), COL (π = 5.5 x 10^−4^), and MEX (π = 6.2 x 10^−4^) was similar to that in BRA (π = 5.6 x 10^−4^), showing that the local BRA population was as diverse as geographically heterogeneous sample sets from other American countries. Similar levels of genome-wide nucleotide diversity were recently reported in *P*. *vivax* populations from Colombia (π = 6.8 x 10^−4^; n = 8) [35], Thailand (π = 5.3 x 10^−4^; n = 88), Cambodia (π = 5.0 x 10^−4^; n = 19), and Indonesia (π = 5.0 x 10^−4^; n = 41) [14], but the differences across studies in genome sequencing depth and criteria for defining high-quality SNPs limit such comparisons.

The frequency distribution of π values within 1-kb sequence windows across the genome was quite similar in all countries (S4 Fig); the interquartile ranges (IQR) were 1.7 x 10^−4^ to 6.9 x 10^−4^ in BRA, 1.7 x 10^−4^ to 6.5 x 10^−4^ in PER, 1.3 x 10^−4^ to 6.6 x 10^−4^ in COL, and 1.9 x 10^−4^ to 7.5 x 10^−4^ in MEX. All distributions were right-skewed, with asymmetry coefficients ranging between 3.37 (BRA) and 3.84 (MEX).

We defined domains with the top 50 π values in a population as highly variable genomic regions. They comprised, in addition to numerous sequences coding for hypothetical proteins, gene families such as *pst-a*, *fam-b*, *fam-d*, and *fam-e* [40] and those coding for major parasite antigens, such as the *vir* family (>300 genes, mostly in subtelomeric domains, on several chromosomes), the serine repeat antigen (*sera*) family (13 genes on chromosome 4), the merozoite surface protein (*msp*)-*7* family (11 genes on chromosome 12), and the *msp-3* family (11 genes on chromosome 10) (S2 Table) (see also [11,14,36]).

These findings are not unexpected, since natural selection favors increased diversity in antigen-coding genes to evade host immunity, but must be interpreted with caution because misalignments of paralogous sequences may have inflated nucleotide diversity estimates in gene families. We thus recalculated genome-wide π values after masking out subtelomeric domains and the internal chromosomal regions comprising the *sera*, *msp-3* and *msp-7* gene families [14], but this procedure affected our overall estimates very little; recalculated values were: BRA (π = 5.5 × 10^−4^), PER (π = 5.2 × 10^−4^), COL (π = 4.7 × 10^−4^), and MEX (π = 5.2 × 10^−4^). The single-copy *msp-1* gene [41] also mapped to a highly variable genomic region in BRA. However, our nucleotide diversity estimates for *msp-1* may have been affected by likely sequence misalignments in the numerous repetitive domains across this locus [42,43].

### Signatures of selection and population expansion

**Fig 3:**
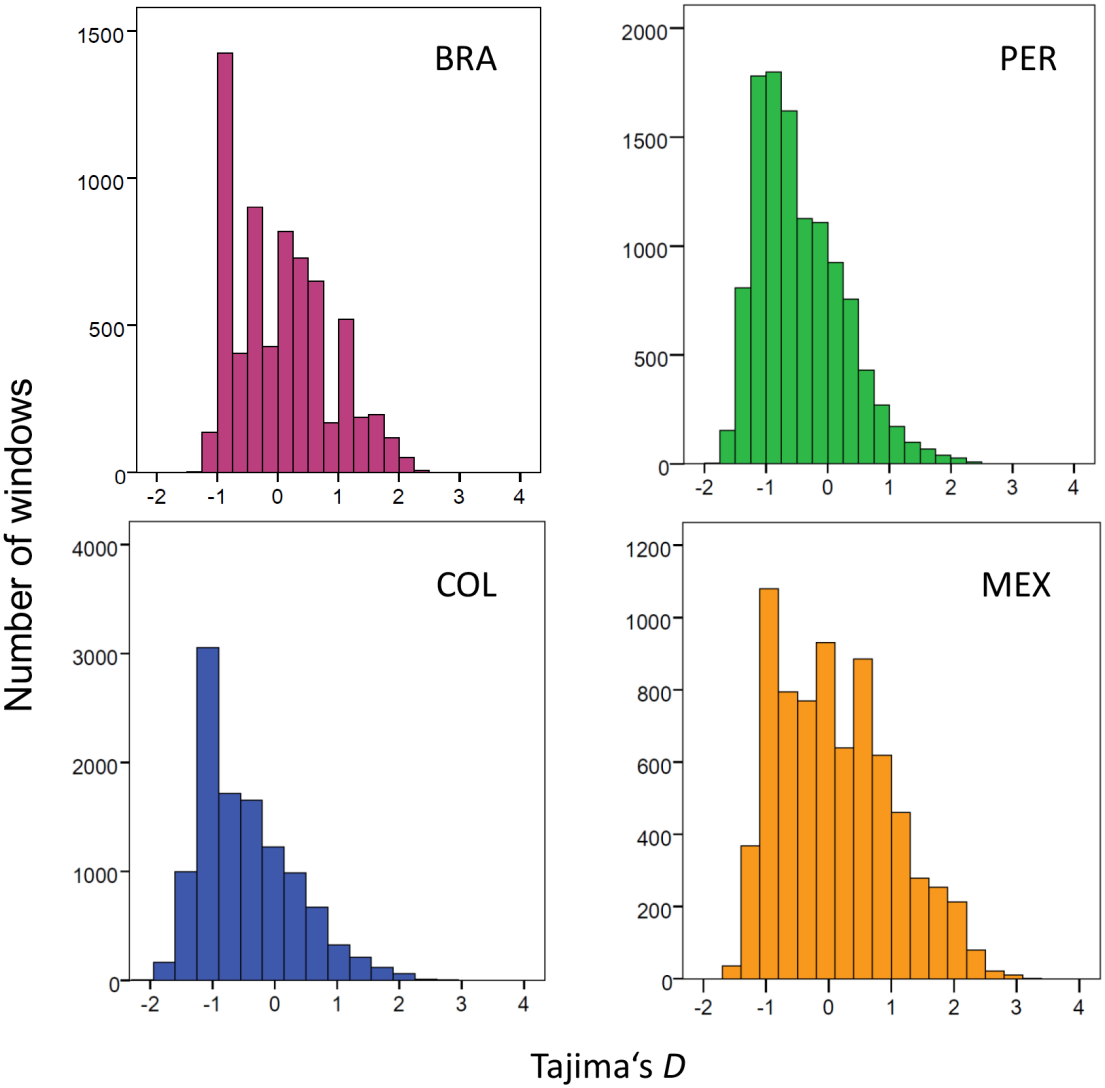
Frequency distribution of Tajima’s *D* values calculated within 1-kb windows of genomic sequence in four New World populations of *P*. *vivax*. BRA = Brazil (n = 11 isolates), PER = Peru (n = 23), COL = Colombia (n = 31), and MEX = Mexico (n = 19).

The expected value for Tajima’s *D* is zero under a neutral model that assumes random mating, no recombination, mutation-drift equilibrium, infinite sites, and constant population size. High Tajima’s *D* values are usually due to balancing selection or recent population size reduction, while negative *D* values are consistent with population size expansion or purifying selection [44]. Although the *P*. *vivax* genomes from COL and PER were not part of a single population (while BRA and MEX genomes are), the Tajima’s *D* distribution provides information on how the pattern of mutations changes across the genome. All distributions of Tajima’s *D* values in our populations were right-skewed, with asymmetry coefficients of 0.477 (BRA), 0.841 (PER), 0.902 (COL), and 0.412 (MEX) (Fig 3). Negative *D* values predominated in PER (median: -0.439; IQR: -0.959 to -0.003) and COL (median: -0.466; IQR: -0.959 to 0.039), but not in BRA (median: 0.026; IQR: -0.571 to 0.595) and MEX (median: 0.039; IQR: -0.724 to 0.748).

Interestingly, the genomic regions with the 50 lowest Tajima’s *D* values within each population (threshold *D* values: -1.096 in BRA, -1.622 in PER, -1.724 in COL, and -1.376 in MEX) comprised several hypothetical and housekeeping genes, but also members of gene families such as *vir*, *sera*, *msp-3*, *pst-a*, and *fam-a* (S3 Table). Only two regions with low Tajima's *D* values were shared by two populations; one had no annotated gene and the other had a gene coding for a hypothetical protein (S3 Table).

Similarly, the genomic regions with the 50 highest Tajima’s *D* values within each population (cut-off values: 2.024 in BRA, 1.890 in PER, 2.078 in COL, and 2.407 in MEX) also comprised several hypothetical and housekeeping genes and a few surface antigen genes (*sera*, circumsporozoite protein [*csp*], and *msp-3*) that may be under balancing selection (S4 Table). However, different genomic regions giving top 50 Tajima’s *D* values were typically found in each population and only 11 of them (none comprising antigen-coding genes) were shared by two or more populations. Whether the high Tajima’s *D* values found in certain domains are due to random effects of the parasite's demographic history or to balancing selection on specific genes remains to be further examined in larger population samples.

MAF distributions were L-shaped in all New World *P*. *vivax* populations (S5 Fig), similar to patterns described for *P*. *falciparum* populations from sub-Saharan Africa [45,46]. The proportion of SNPs with allele frequencies ≤ 0.1 were 51.6% in BRA, 66.3% in PER, 71.5% in COL, and 55.1% in MEX. Despite the relatively small sample sizes, we interpret the clear predominance of negative Tajima’s *D* values in PER and COL and of rare alleles in all populations, but mainly in PER and COL, as suggestive of a recent *P*. *vivax* population expansion in the Americas. Data from an extensive mitochondrial genome analysis of local parasites are also consistent with the demographic expansion hypothesis [47].

### Genetic differentiation and population structure

**Fig 4:**
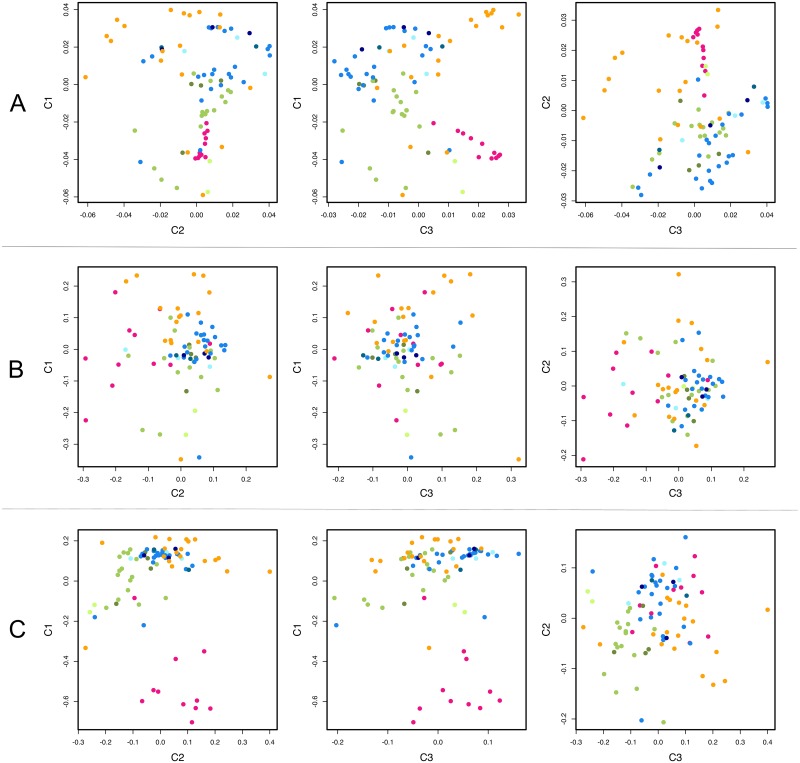
Principal component analysis of the population structure of *P*. *vivax* in the New World. Panel A shows results using the complete data set of 94,122 SNPs; panel B shows results obtained with a set of 37 segregating SNPs included in a previously described *P*. *vivax* barcode [49]; and panel C shows results obtained with a hypothetical barcode comprising the 100 SNPs yielding the highest average Wright’s fixation index *F*_ST_ values in pairwise comparisons of New World *P*. *vivax* populations. The SNPs used in the analysis shown in panel C are listed in S5 Table. Samples are colored according to their geographic origin as shown in Fig 1. The percentage contributions of each principal component (C1, C2, and C3) to overall variance were as follows: panel A, C1 = 22.27%, C2 = 14.15%, and C3 = 8.15%; panel B, C1 = 19.93%, C2 = 12.03%, and C3 = 11.42%; and panel C, C1 = 21.36%, C2 = 10.93%, and C3 = 9.33%.

*P*. *vivax* genomic sequences from the New World were previously shown to cluster mostly according to their geographic origins, with the majority of MEX samples (that show more extensive evidence of identity by descent) clustering together in PCA plots [11]. These findings are particularly relevant to malaria-eliminating countries in the continent; if parasites can be assigned by molecular genotyping to their countries of origin, locally acquired and imported infections can theoretically be easily distinguished. Moreover, genetic analysis can also help to determine the likely origin of imported infections [10,48]. Accordingly, our PCA data revealed a clear clustering of BRA samples, which were separated from other populations by principal components C1 and C3 when the complete SNP data set was used. MEX was separated from other populations mostly by C1 and C2, while PER and COL were less clearly differentiated (Fig 4A).

**Fig 5:**
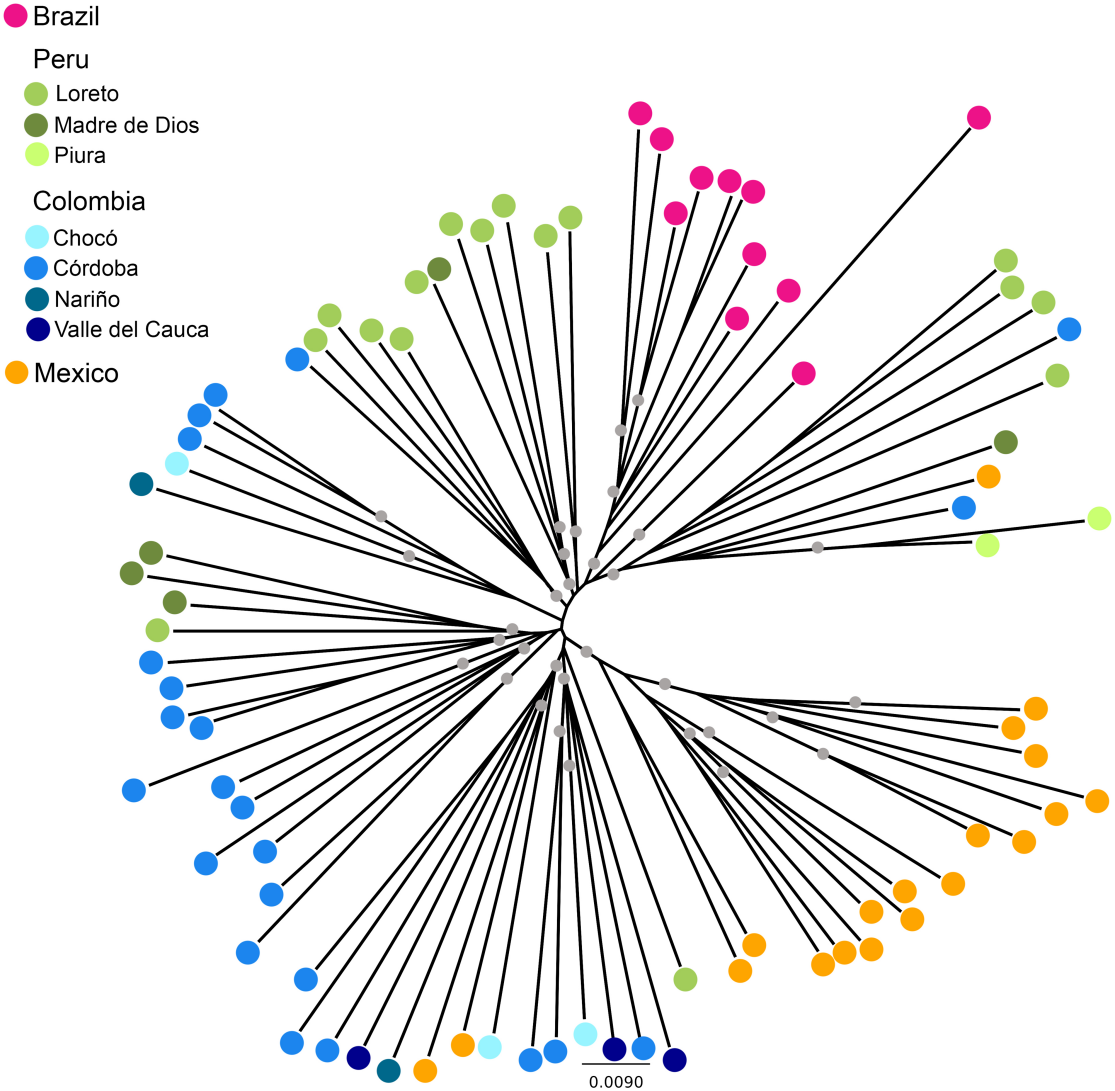
A neighbor-joining phylogenetic tree with 1,000 bootstrap pseudoreplicates obtained with genomic sequences of *P*. *vivax* isolates from Brazil (n = 11 isolates), Peru (n = 23), Colombia (= 31), and Mexico (n = 19). Branch tips were colored according to the geographic origin of samples, as shown in Fig 1. Grey circles indicate internal nodes with > 70% bootstrap support.

Moreover, a rather similar sample clustering pattern was revealed by a standard, neighbor-joining phylogenetic analysis (Fig 5). All BRA samples and most (16 of 19) MEX samples formed well-supported clades, while PER and COL isolates spread across single- and multiple-country clades.

Similar to PCA and phylogenetic analysis, the model-based clustering approach implemented in ADMIXTURE software [33] also defined BRA and MEX as distinct populations (S6A Fig, number of clusters *K* = 3) based on the whole genomic data set. PER and COL were nearly indistinguishable. A similar analysis with 2 clusters did not differentiate MEX from COL (S6A Fig, number of clusters *K* = 2). ADMIXTURE analysis using a curtailed data set (12,762 unlinked SNPs) allowed a poor differentiation between populations (S6B Fig, number of clusters *K* = 2 or 3). Not surprisingly, the most geographically homogeneous samples (BRA and MEX) formed well-defined clusters.

We next tested whether a recently described *P*. *vivax* SNP barcode [49] would be able to correctly assign New World isolates to their countries of origin. Of the 42 SNPs originally included in the barcode, five did not segregate in our populations. PCA with the remaining 37 SNPs was unable to cluster our parasite populations by their countries of origin (Fig 4B).

The overall pairwise differentiation between populations, estimated using *F*_ST_ averaged across the entire genome, was directly proportional to the geographic distance between sites, being lowest between COL and PER and highest between BRA and MEX (Table 2). Among the 100 SNPs with the highest average pairwise *F*_ST_ estimates, only two mapped to antigen-coding genes (both to *sera* gene paralogs on chromosome 4); all others were either noncoding SNPs or mapped to genes encoding hypothetical or housekeeping proteins (S5 Table). We next tested whether these 100 SNPs with the top *F*_ST_ values could separate New World populations more efficiently than the complete genome-wide SNP set (compare Fig 4A and 4C) and the currently available 42-SNP *P*. *vivax* barcode [49] (compare Fig 4B and 4C). These results suggest that regional SNP barcodes could be further explored to track the geographic origin of *P*. *vivax* samples in the Americas.

**Table 2:** Pairwise differentiation between populations, estimated using the Wright's fixation index *F*_ST_ averaged across all loci, in *Plasmodium vivax* populations from Brazil, Peru, Colombia, and Mexico.

Country	Brazil	Peru	Colombia	Mexico
**Brazil**		0.037	0.055	0.092
**Peru**			0.030	0.047
**Colombia**				0.025

### Nucleotide substitutions and antimalarial drug resistance

Our genomic sequence data enabled the identification of SNPs mapping to several *P*. *vivax* genes known or suspected to be involved in antimalarial drug resistance (Table 3). We found nonsynonymous mutations at the dihydrofolate reductase-thymidylate synthase (*dhfr*) locus previously associated with pyrimethamine resistance (S58R and S117N) almost exclusively in COL (see also [35]) and PER (see also [37]), whereas antifolates are no longer recommended in these countries; two previously undescribed SNPs (H99N and H99R) were also found in COL. The dihydropteroate synthase (*dhps*) SNP A383G, associated with sulfadoxine resistance, was also observed in COL and PER (see also [35]), with the M205I change being found only in PER (see also [36]). Little or no polymorphism in *dhfr* and *dhps* genes was observed in BRA and MEX, likely reflecting differences in sulfadoxine-pyrimethamine use across countries in the region. Although pyrimethamine and sulfadoxine has never been recommended as a treatment for *P*. *vivax* infection, they may have been used to treat patients co-infected with *P*. *falciparum* and *P*. *vivax*, exposing the latter parasite to these drugs and likely selecting for resistant phenotypes. Moreover, the frequent use of sulfonamides to treat co-occurring, unrelated (mostly bacterial) infections may have selected sulfa-resistant strains in malaria parasite carriers from some of these countries.

**Table 3:** Nonsynonymous single-nucleotide polymorphism (SNPs) detected in drug resistance genes by whole-genome sequencing in *Plasmodium vivax* isolates from Brazil, Peru, Colombia, and Mexico.

Gene	Chromosome	Amino acid change	No. (%) samples with SNP per country			
			Brazil	Peru	Colombia	Mexico
***dhfr* (PVX_089950)**	5	S58R	0 (0.0%)	2 (8.7%)	5 (16.1%)	0 (0.0%)
		H99N	0 (0.0%)	0 (0.0%)	2 (6.4%)	0 (0.0%)
		H99R	0 (0.0%)	0 (0.0%)	2 (6.4%)	0 (0.0%)
		S117N	1 (9.1%)	4 (17.4%)	17 (54.8%)	0 (0.0%)
***dhps* (PVX_123230)**	14	M205I	0 (0.0%)	1 (4.3%)	0 (0.0%)	0 (0.0%)
		A383G	0 (0.0%)	1 (4.3%)	1 (3.2%)	0 (0.0%)
***mdr1* (PVX_080100)**	10	L186W	0 (0.0%)	1 (4.3%)	0 (0.0%)	0 (0.0%)
		V221L	0 (0.0%)	2 (8.7%)	0 (0.0%)	0 (0.0%)
		M908L	0 (0.0%)	8 (34.8%)	3 (9.7%)	0 (0.0%)
		T958M	0 (0.0%)	9 (39.1%)	6 (19.3%)	4 (21.0%)
		Y976F	1 (9.1%)^a^	0 (0.0%)	0 (0.0%)	0 (0.0%)
		F1070L	0 (0.0%)	2 (8.7%)	0 (0.0%)	0 (0.0%)
		F1076L	1 (9.1%)^a^	1 (4.3%)	0 (0.0%)	0 (0.0%)
***mrp1* (PVX_097025)**	2	T259R	0 (0.0%)	1 (4.3%)	13 (56.5%)	12 (63.2%)
		T282M	0 (0.0%)	0 (0.0%)	5 (16.1%)	0 (0.0%)
		K542E	0 (0.0%)	0 (0.0%)	1 (3.2%)	0 (0.0%)
		Q906E	0 (0.0%)	7 (30.4%)	9 (29.0%)	0 (0.0%)
		L1282I	0 (0.0%)	0 (0.0%)	2 (6.4%)	0 (0.0%)
		Y1393D	3 (27.3%)^b^	8 (34.8%)	0 (0.0%)	2 (10.5%)
		G1419A	1 (9.1%)^c^	5 (21.8%)	2 (6.4%)	1 (5.3%)
		V1478I	2 (18.2%)^d^	4 (17.4%)	1 (3.2%)	1 (5.3%)
		I1480V	0 (0.0%)	0 (0.0%)	1 (3.2%)	0 (0.0%)
		T1525I	0 (0.0%)	0 (0.0%)	1 (3.2%)	0 (0.0%)
		H1586I	0 (0.0%)	4 (17.4%)	0 (0.0%)	0 (0.0%)
***mrp2* (PVX_124085)**	14	S1701L	0 (0.0%)	1 (4.3%)	0 (0.0%)	0 (0.0%)
		T1698K	0 (0.0%)	2 (8.7%)	0 (0.0%)	5 (26.5%)
		P1600H	0 (0.0%)	0 (0.0%)	0 (0.0%)	1 (5.3%)
		Q1419E	0 (0.0%)	2 (8.7%)	3 (9.7%)	3 (6.9%)
		N1263Y	0 (0.0%)	2 (8.7%)	1 (3.2%)	0 (0.0%)
		P1196S	0 (0.0%)	0 (0.0%)	1 (3.2%)	4 (21.0%)
		G1166E	0 (0.0%)	0 (0.0%)	0 (0.0%)	5 (26.5%)
		A1106S	0 (0.0%)	0 (0.0%)	3 (9.7%)	7 (37.1%)
		V1022M	1 (9.1%)	4 (17.4%)	2 (6.4%)	0 (0.0%)
		S681I	0 (0.0%)	0 (0.0%)	5 (16.1%)	0 (0.0%)
		R294M	1 (9.1%)	3 (12.9%)	16 (51.2%)	12 (63.6%)

^a^The sample from Brazil carrying these two mutations (#18) was not tested for chloroquine resistance.

^b^Two of three samples (#20 and #207) carrying this mutation were characterized as chloroquine-sensitive using an ex-vivo schizont maturation test (IC_50_ = 14.5 and 46.7 mM, respectively); the third sample (#Brazil32) [11] was not tested for chloroquine resistance.

^c^The sample from Brazil carrying this mutation (#207) was characterized as chloroquine-sensitive (IC_50_ = 46.7 mM)

^d^One sample from Brazil carrying this mutation (#32) was characterized as chloroquine-sensitive (IC_50_ = 20.5 mM); the second sample (#Brazil32) [11] was not tested for chloroquine resistance.

Non-synonymous substitutions were also characterized in the multidrug resistance 1 protein (*mdr1*) gene, including two previously undescribed (L186W and F1070L) and six previously reported SNPs (V221L, M908L, T958M, Y976F, and F1076L [50]). The Y976F change, originally believed to confer chloroquine resistance (CQR) in Southeast Asia and Melanesia [24], occurred in a single BRA sample whose CQR phenotype could not be determined (Table 2). Interestingly, we found no coding changes in the chloroquine resistance transporter ortholog (*crt-o*) gene (PVX 087980), whose ortholog in *P*. *falciparum* displays a key non-synonymous substitution leading to CQR [51].

Five of 12 mutations found at the multidrug resistance associated protein 1 (*mrp1* [PVX 097025]) locus, which codes for an ATP-cassette binding (ABC) transporter putatively involved in antimalarial drug efflux [52], had been described in a clinical sample from Peru (L1282I, Y1393D, G1419A, V1478I, and H1586I [36]). Similarly, six of 11 had been previously described in Colombia (Q1419E, P1196S, A1106S, V1022M, S681I, R294M [35]) and six in Peru (S1701L, T1698K, Q1419E, A1106S, V1022M, and R294M [37]), in multidrug resistance associated protein 2 gene (PVX_124085). The antimalarial drug-resistance phenotype (if any) associated with these mutations remains undetermined.

### Linkage disequilibrium

**Fig 6:**
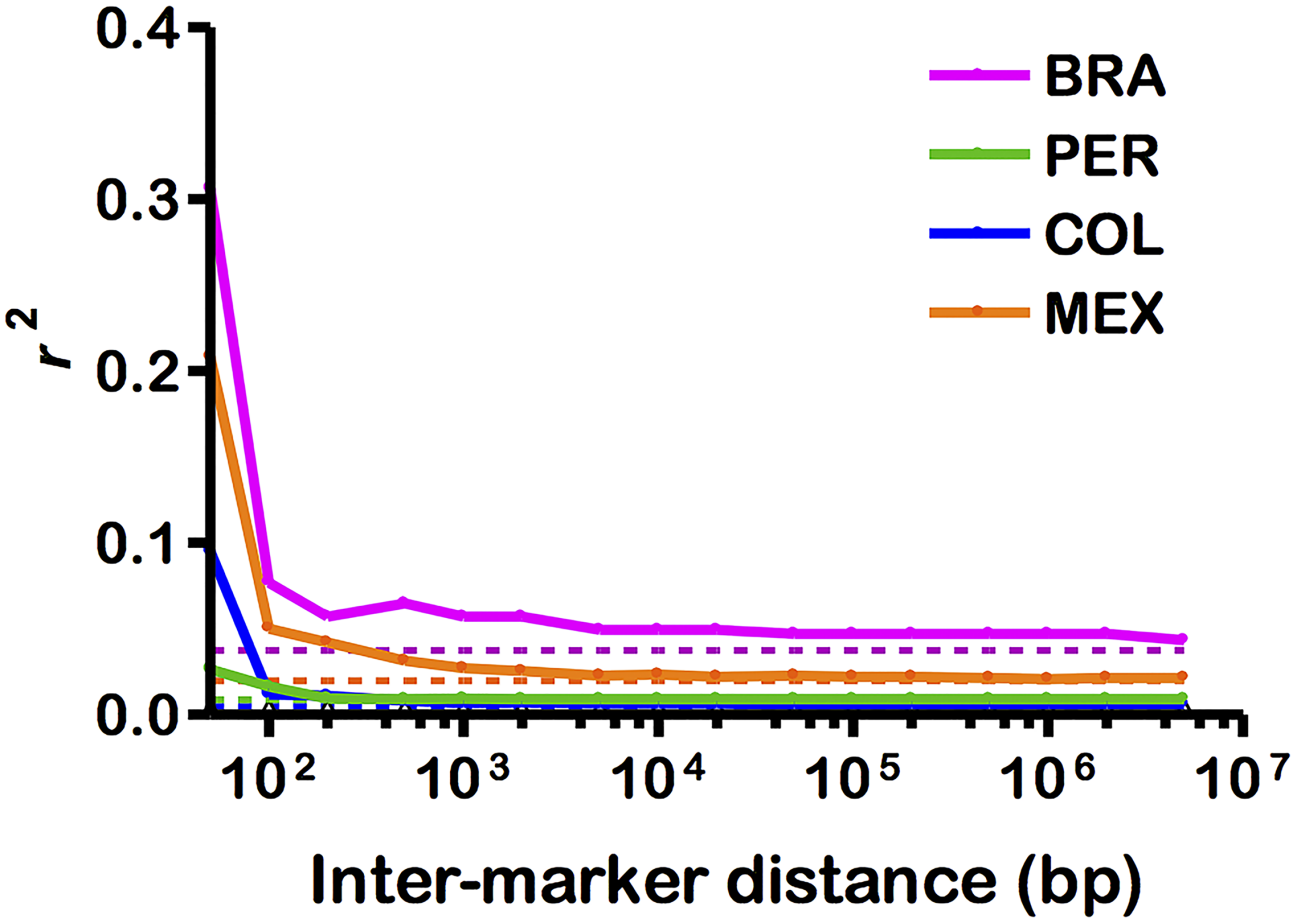
Linkage disequilibrium (LD) decay with increasing distance between SNPs on the same chromosome. Solid lines show median *r*^2^ estimates for SNPs along the same chromosome plotted against the inter-marker physical distance for Brazil (pink), Peru (green), Colombia (blue), and Mexico (orange). Dotted lines show the background genome-wide LD (median *r*^2^ estimates for unlinked SNPs—those mapping to different chromosomes).

Here we found a sharp LD decline within 100 bp of distance between pairs of SNPs in BRA and MEX, with very low LD in COL and negligible LD in PER (Fig 6). Interestingly, the LD decay in BRA and MEX populations, originating from the sites with the lowest malaria transmission [53], was even faster than that described for *P*. *vivax* populations from Southeast Asia, where *P*. *vivax* endemicity is substantially higher [14], and that described for *P*. *falciparum* populations from areas of much higher endemicity in Sub-Saharan Africa [54, 55, 56] and Southeast Asia [56]. These results are consistent with a very high outcrossing rate in *P*. *vivax* populations and imply that SNP array-based genome-wide association studies (GWAS) would require a very high marker density (inter-marker distance < 100 bp) to help find genetic loci that determine phenotypes of interest, such as drug resistance and virulence, in BRA and MEX populations. SNP-based association studies are unlikely to be successful in PER and COL populations, given the negligible LD. In contrast, *P*. *falciparum* populations from the Pacific Coast of Colombia showed a much more gradual LD decline, reaching background LD levels only at an inter-marker distance of 240 kb [57].

### Conclusions

We found high genome-wide diversity and relatively little geographic structure in *P*. *vivax* populations from areas of relatively low malaria transmission in the Americas. These findings contrast with the low genetic diversity and clear subdivision into local, highly divergent populations that is typical of New World populations of *P*. *falciparum* [57–61]. Interestingly, local *P*. *vivax* populations are nearly as polymorphic as their *P*. *falciparum* counterparts from Africa [45,46,54] and their *P*. *vivax* counterparts from Southeast Asia [11,14]. Moreover, the low levels of between-population differentiation in *P*. *vivax* from the Americas are reminiscent of those in *P*. *falciparum* populations from hyperendemic sub-Saharan Africa [45,46,59, 62].

Demographic history might explain, at least in part, the observed differences between *P*. *vivax* and *P*. *falciparum* populations from the New World. The original diversity of the ancestral *P*. *falciparum* population may have been partially lost during (a) its migration to the Americas in post-Columbian times [63] followed by the adaptation to entirely different local vectors [64], (b) recent selective sweeps driven by antimalarial drugs, such as chloroquine [65] and pyrimethamine [66], and (c) local extinctions and clonal expansions following eradication attempts in the Americas between the mid-1950s and late 1960s [53,60]. These events would have resulted in small and fragmented, mostly inbred *P*. *falciparum* populations being scattered throughout the region. The significantly greater genome-wide diversity currently found in *P*. *vivax* populations worldwide, compared with *P*. *falciparum* [11,67], suggests that (a) this species retained more ancestral diversity than *P*. *falciparum* when transferred from apes to humans and following subsequent spread out of Africa and/or (b) *P*. *vivax* is simply older than *P*. *falciparum* and therefore has accumulated more mutations over time. Moreover, these species might also differ in the ways they colonized the New World. The high diversity of present-day *P*. *vivax* lineages across this continent is consistent with an admixture of parasite lineages originating from geographically diverse regions [5, 7, 47], including now extinct European lineages [12]. The success of colonization events and the recombination between these introduced strains may have been favored by the ability of *P*. *vivax* to stay dormant in the liver as hypnozoites, prolonging the duration of parasite carriage in human hosts and increasing the probability of superinfections leading to the co-occurrence of genetically diverse clones that may recombine once transmitted to mosquito vectors. Moreover, genome-wide *P*. *vivax* diversity in the region appears to have been little affected by recent selective sweeps driven by pyrimethamine [35,37] in Brazil and Colombia. Interestingly, *P*. *vivax* resistance to chloroquine remains relatively infrequent in the New World [68] and is unlikely to have induced a major selective sweep in local parasites.

Improved methods for clinical sample preparation and next-generation sequencing now enable further genome-wide analyses of additional *P*. *vivax* isolates from the New World, to test the demographic scenarios suggested by our data.

## Supporting information

S1 Fig Single-step filtering procedure used to remove leukocytes from clinical blood samples in a field laboratory. *A*, BioR 01 Plus leukocyte depletion filter (Fresenius Kabi, Bad Homburg, Germany). *B* and *C*, cutting off with a scissor, under sterile conditions, the tubing connecting the filtering device to the 400-ml blood storage bag and the adapter. *D*, use of filtering device in a laminar flow hood, indicating how the blood sample is introduced and collected, with 10-ml syringes, after filtering.
(PDF)

S2 Fig Cross-validation errors estimated for Admixture analysis under *K* values ranging between 2 and 10. Panel A shows results for analysis comprising all 94,122 high-quality SNPs, while Panel B shows those for the curtailed set of 12,762 SNPs that are not linked.
(PDF)

S3 Fig Enrichment of *P*. *vivax* DNA isolated from clinical samples using the single-step filtering procedure described in S1 Fig. Fold increase in parasite:human DNA ratio after leukocyte depletion is shown in relation to the initial, pre-filtering parasite:human DNA ratio. Data on *x* and *y* axes are shown in log scale.
(PDF)

S4 Fig Frequency distribution of nucleotide diversity (π) calculated within 1-kb windows of genomic sequence in four New World populations of *P*. *vivax*. BRA = Brazil (n = 11 isolates), PER = Peru (n = 23), COL = Colombia (n = 31), and MEX = Mexico (n = 19).
(PDF)

S5 Fig Relative frequency (%) distribution of minor allele frequencies (MAF) in four New World populations of *P*. *vivax*. BRA = Brazil (n = 11 isolates), PER = Peru (n = 23), COL = Colombia (n = 31), and MEX = Mexico (n = 19).
(PDF)

S6 Fig Population structure evaluated using a model-based clustering approach implemented in the software Admixture [34]. We show Admixture plots obtained with *K* = 2 and *K* = 3 clusters with all 94,122 high-quality SNPs (panel A) or a curtailed set of 12,762 SNPs that are not linked (panel B). Although *K* = 2 was associated with the lowest cross-validation error (S4 Fig), the analysis of all high-quality SNPs under *K* = 3 appeared more able to separate BRA and MEX from the other populations. The analysis with the curtailed SNP set yielded less clear differentiation among populations.
(PDF)

S1 Table *P*. *vivax* sequence data sets from the New World analyzed in this study. Isolate codes, country of origin, and Sequence Read Archive (SRA) accession numbers are provided.
(PDF)

S2 Table List of annotated genes mapping to the 50 windows (1 kb) with the highest π values in each New World *P*. *vivax* population. (PDF)

S3 Table List of annotated genes mapping to the 50 windows (1 kb) with the lowest Tajima's *D* values in each New World *P*. *vivax* population. (PDF)

S4 Table List of annotated genes mapping to the 50 windows (1 kb) with the highest Tajima's *D* values in each New World *P*. *vivax* population. (PDF)

S5 Table List of SNPs with the 100 highest average Wright’s fixation index *F*_ST_ values in pairwise comparisons of New World *P*. *vivax* populations. (PDF)
