# Supplementary material for: Genome-wide diversity and differentiation in New World populations of the human malaria parasite Plasmodium vivax
Source: PLoS Negl Trop Dis. 2017 Jul 31;11(7):e0005824. doi: 10.1371/journal.pntd.0005824 (PMC5552344; doi:10.1371/journal.pntd.0005824)
Supplement: S1 Fig — A, BioR 01 Plus leukocyte depletion filter (Fresenius Kabi, Bad Homburg, Germany). B and C, cutting off with a scissor, under sterile conditions, the tubing connecting the filtering device to the 400-ml blood storage bag and the adapter. D, use of filtering device in a laminar flow hood, indicating how the blood sample is introduced and collected, with 10-ml syringes, after filtering. (PDF) [file pntd.0005824.s001.pdf]

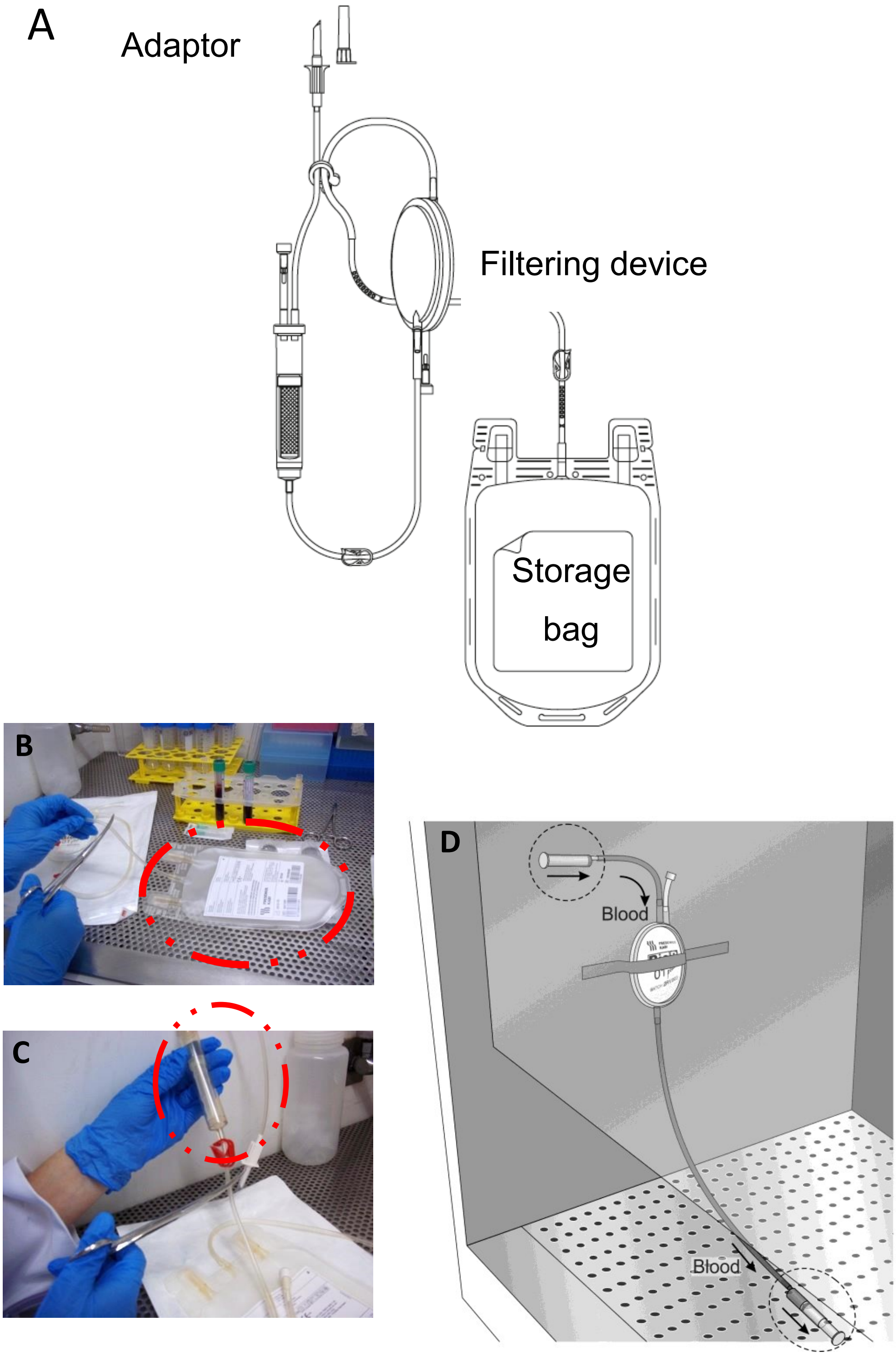

S1 Fig. Single-step filtering procedure used to remove leukocytes from clinical blood samples in a field laboratory. *A*, BioR 01 Plus leukocyte depletion filter (Fresenius Kabi, Bad Homburg, Germany). *B* and *C*, cutting off with a scissor, under sterile conditions, the tubing connecting the filtering device to the 400-ml blood storage bag and the adapter. *D*, use of filtering device in a laminar flow hood, indicating how the blood sample is introduced and collected, with 10-ml syringes, after filtering.
