# Supplementary material for: Genome-wide diversity and differentiation in New World populations of the human malaria parasite Plasmodium vivax
Source: PLoS Negl Trop Dis. 2017 Jul 31;11(7):e0005824. doi: 10.1371/journal.pntd.0005824 (PMC5552344; doi:10.1371/journal.pntd.0005824)
Supplement: S2 Fig — Panel A shows results for analysis comprising all 94,122 high-quality SNPs, while Panel B shows those for the curtailed set of 12,762 SNPs that are not linked. (PDF) [file pntd.0005824.s002.pdf]

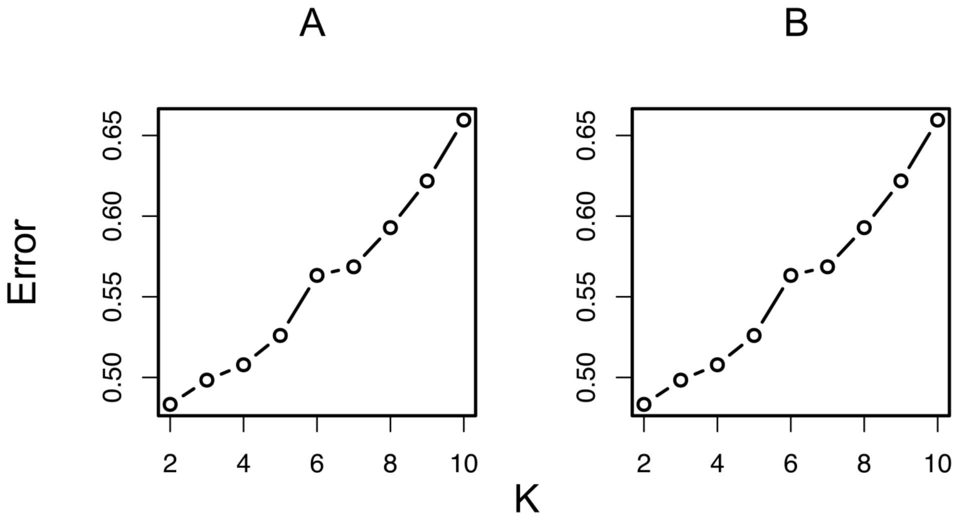

S2 Fig. Cross-validation errors estimated for Admixture analysis under *K* values ranging between 2 and 10. Panel A shows results for analysis comprising all 94,122 high-quality SNPs, while Panel B shows those for the curtailed set of 12,762 SNPs that are not linked.
