# Supplementary material for: Genome-wide diversity and differentiation in New World populations of the human malaria parasite Plasmodium vivax
Source: PLoS Negl Trop Dis. 2017 Jul 31;11(7):e0005824. doi: 10.1371/journal.pntd.0005824 (PMC5552344; doi:10.1371/journal.pntd.0005824)
Supplement: S3 Fig — Fold increase in parasite:human DNA ratio after leukocyte depletion is shown in relation to the initial, pre-filtering parasite:human DNA ratio. Data on x and y axes are shown in log scale. (PDF) [file pntd.0005824.s003.pdf]

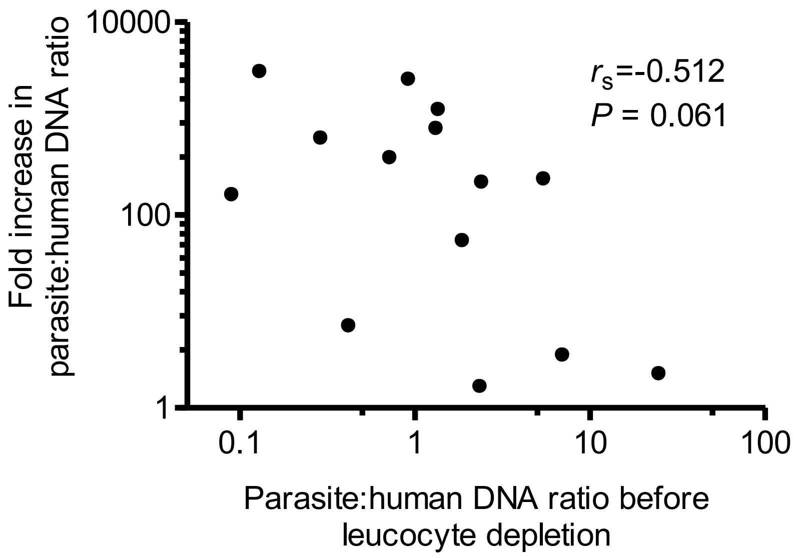

S3 Fig. **Enrichment of *P. vivax* DNA isolated from clinical samples using the single-step filtering procedure described in S2 Fig**. Fold increase in parasite:human DNA ratio after leukocyte depletion is shown in relation to the initial, pre-filtering parasite:human DNA ratio. Data on *x* and *y* axes are shown in log scale.
