# Supplementary material for: Genome-wide diversity and differentiation in New World populations of the human malaria parasite Plasmodium vivax
Source: PLoS Negl Trop Dis. 2017 Jul 31;11(7):e0005824. doi: 10.1371/journal.pntd.0005824 (PMC5552344; doi:10.1371/journal.pntd.0005824)
Supplement: S5 Fig — BRA = Brazil (n = 11 isolates), PER = Peru (n = 23), COL = Colombia (n = 31), and MEX = Mexico (n = 19). (PDF) [file pntd.0005824.s005.pdf]

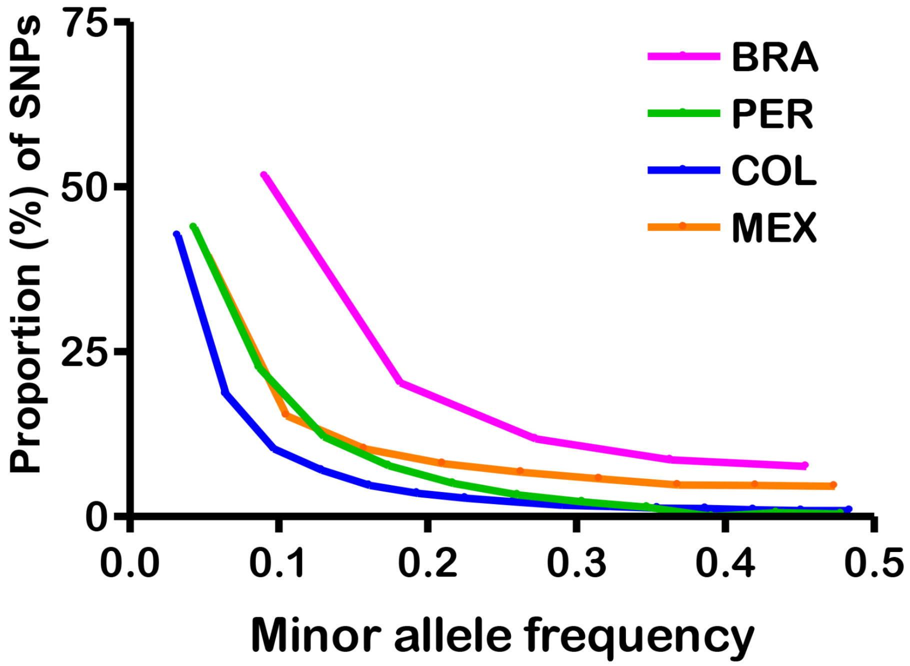

S5 Fig. Relative frequency (%) distribution of minor allele frequencies (MAF) in four New World populations of *P. vivax*. BRA = Brazil (n = 11 isolates), PER = Peru (n = 23), COL = Colombia (n = 31), and MEX = Mexico (n = 19).
