# Supplementary material for: Genome-wide diversity and differentiation in New World populations of the human malaria parasite Plasmodium vivax
Source: PLoS Negl Trop Dis. 2017 Jul 31;11(7):e0005824. doi: 10.1371/journal.pntd.0005824 (PMC5552344; doi:10.1371/journal.pntd.0005824)
Supplement: S6 Fig — We show Admixture plots obtained with K = 2 and K = 3 clusters with all 94,122 high-quality SNPs (panel A) or a curtailed set of 12,762 SNPs that are not linked (panel B). Although K = 2 was associated with the lowest cross-validation error (S4 Fig), the analysis of all high-quality SNPs under K = 3 appeared more able to separate BRA and MEX from the other populations. The analysis with the curtailed SNP set yielded less clear differentiation among populations. (PDF) [file pntd.0005824.s006.pdf]

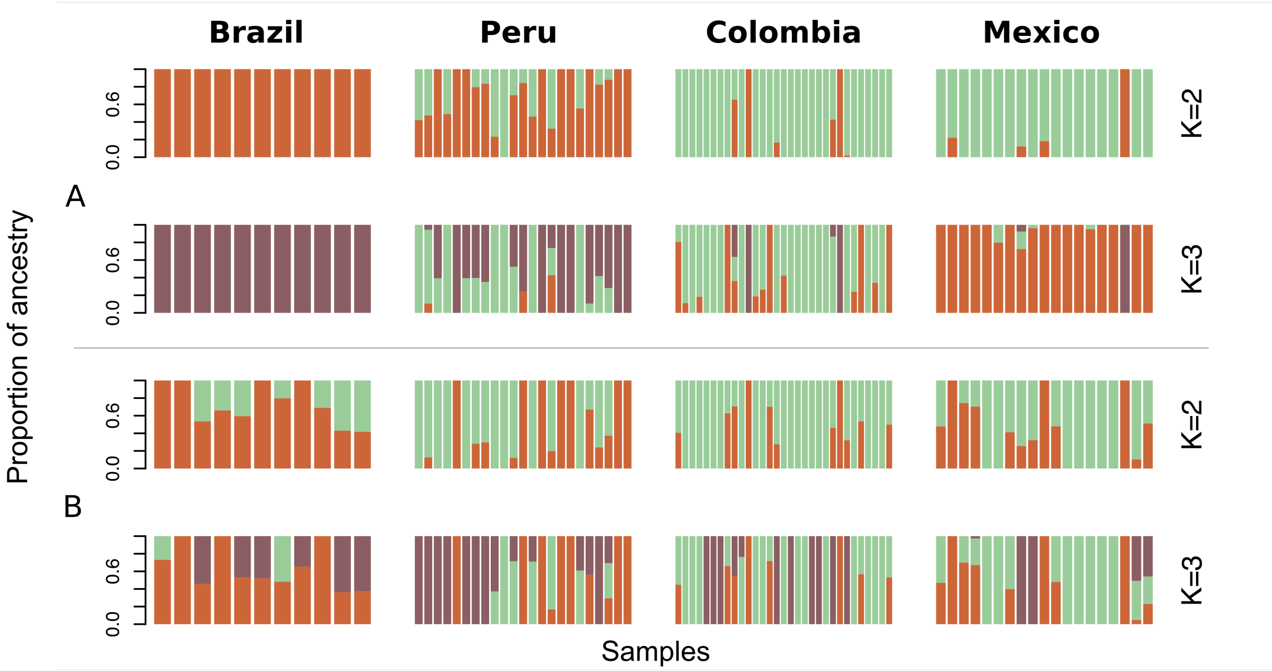

S6 Fig. Population structure evaluated using a model-based clustering approach implemented in the software Admixture [34]. We show Admixture plots obtained with *K* = 2 and *K* = 3 clusters with all 94,122 high-quality SNPs (panel A) or a curtailed set of 12,762 SNPs that are not linked (panel B). Although *K* = 2 was associated with the lowest cross-validation error (S4 Fig), the analysis of all high-quality SNPs under *K* = 3 appeared more able to separate BRA and MEX from the other populations. The analysis with the curtailed SNP set yielded less clear differentiation among populations.
