# Supplementary material for: Genome-wide diversity and differentiation in New World populations of the human malaria parasite Plasmodium vivax
Source: PLoS Negl Trop Dis. 2017 Jul 31;11(7):e0005824. doi: 10.1371/journal.pntd.0005824 (PMC5552344; doi:10.1371/journal.pntd.0005824)
Supplement: S1 Table — Isolate codes, country of origin, and Sequence Read Archive (SRA) accession numbers are provided. (PDF) [file pntd.0005824.s007.pdf]

S1 Table. *P. vivax* sequence data sets from the New World analyzed in this study. Isolate codes, country of origin, and Sequence Read Archive (SRA) accession numbers are provided.

Country	Sample	SRA Access Number
Brazil	17	SRR5098034
	18	SRR5099324
	19	SRR5099325
	20	SRR5099326
	32	SRR5278293
	51	SRR5278295
	52	SRR5278298
	207	SRR5278291
	ACR	SRR5278301
	PV4	ERR019040
	Brazil32	SRR1568192
Colombia	Col015	SRR1564664
	Col020	SRR1568235
	Col026	SRR1568128
	Col036	SRR1573226
	Col038	SRR1562524
	Col040	SRR1568221
	Col280	SRR1568236
	Col295	SRR1562967
	Col437	SRR1568171
	Col438-A	SRR1568155
	Col438-B	SRR1568227
	Col439	SRR1562975
	Col440	SRR1568230
	Col441-A	SRR1568159
	Col441-B	SRR1567977
	Col443-A	SRR1562971
	Col443-B	SRR1562555
	Col445	SRR1568207
	Col446	SRR1568118

	Col448	SRR1564670
	Col449	SRR1562870
	Col485	SRR1568213
	Col486	SRR1568160
	Col487	SRR1562518
	Col488	SRR1564665
	Col489	SRR1564660
	Col490	SRR1568169
	Col491	SRR1564650
	Col492	SRR1562965
	Col493	SRR1562818
	Col504	SRR1568112
**Peru**	Peru06	SRR1568172
	Peru09	SRR1562513
	Peru10	SRR1562521
	Peru257	SRR1568149
	Peru258	SRR1562871
	Peru259	SRR1562958
	Peru260	SRR1568196
	Peru262	SRR1568174
	Peru00622	SRR1568166
	Peru00699	SRR1568216
	Peru852	SRR1568182
	Peru858	SRR1568195
	Peru869	SRR1562534
	Peru1008	SRR1568206
	Peru1021	SRR1562567
	Peru1022	SRR1562851
	Peru2025	SRR1568162
	Peru3133	SRR1564630
	Peru3136	SRR1568157
	Peru3232	SRR1568232
	Peru3280	SRR1562615
	Peru4017	SRR1562538

	Peru4023	SRR1568117
Mexico	Mexico330-A	SRR1568231
	Mexico267-A	SRR1568225
	Mexico980-A	SRR1568223
	Mexico1086-A	SRR1568219
	Mexico55-03	SRR1568218
	Mexico938-A	SRR1568201
	Mexico32-E-03	SRR1568190
	Mexico203-04	SRR1568181
	Mexico118-A	SRR1568158
	Mexico165-A	SRR1568153
	Mexico161-04	SRR1568150
	Mexico760-A	SRR1568127
	Mexico566-A	SRR1568126
	Mexico63-08	SRR1568110
	Mexico21-A	SRR1568077
	Mexico10-E-05	SRR1562840
	Mexico533-07	SRR1562526
	Mexico31A-03	SRR1562839
	Mexico1067-A	SRR1562968
