# Supplementary material for: Genome-wide diversity and differentiation in New World populations of the human malaria parasite Plasmodium vivax
Source: PLoS Negl Trop Dis. 2017 Jul 31;11(7):e0005824. doi: 10.1371/journal.pntd.0005824 (PMC5552344; doi:10.1371/journal.pntd.0005824)
Supplement: S2 Table — (PDF) [file pntd.0005824.s008.pdf]

**S2 Table. List of annotated genes mapping to the 50 windows (1 kb-wide) with the highest nucleotide diversity in each New World P. vivax population**

| Country | Chromosome | Start (bp) | End (bp) | SNPs | Nucleotide Diversity | Gene |
|---|---|---|---|---|---|---|
| Brazil | 1 | 773001 | 774000 | 11 | 0.00386957 | PVX_093680(Phist protein (Pf- |
| Brazil | 2 | 66001 | 67000 | 12 | 0.00378261 | PVX_096965(VIR) |
| Brazil | 3 | 19001 | 20000 | 14 | 0.00336232 | PVX_001080(Hypothetical) |
| Brazil | 3 | 9001 | 10000 | 9 | 0.00405797 | PVX_001100(RAD (Pv-fam-e)) |
| Brazil | 3 | 26001 | 27000 | 18 | 0.00411594 | PVX_001080(Hypothetical) |
| Brazil | 3 | 22001 | 23000 | 13 | 0.00444928 | PVX_001080(Hypothetical) |
| Brazil | 3 | 25001 | 26000 | 15 | 0.00466667 | PVX_001080(Hypothetical) |
| Brazil | 3 | 21001 | 22000 | 19 | 0.00484058 | PVX_001080(Hypothetical) |
| Brazil | 3 | 14001 | 15000 | 17 | 0.00486957 | PVX_001090(Hypothetical) |
| Brazil | 3 | 472001 | 473000 | 11 | 0.00494203 | - |
| Brazil | 4 | 566001 | 567000 | 13 | 0.00352174 | PVX_003840(SERA) |
| Brazil | 4 | 597001 | 598000 | 11 | 0.0035942 | PVX_003805(SERA) |
| Brazil | 4 | 568001 | 569000 | 14 | 0.00386957 | PVX_003835(SERA) |
| Brazil | 4 | 584001 | 585000 | 12 | 0.00407246 | PVX_003820(SERA) |
| Brazil | 4 | 581001 | 582000 | 12 | 0.00417391 | PVX_003825(SERA) |
| Brazil | 4 | 594001 | 595000 | 12 | 0.00453623 | PVX_003810(SERA) |
| Brazil | 4 | 564001 | 565000 | 21 | 0.00676812 | PVX_003840(SERA) |
| Brazil | 5 | 1256001 | 1257000 | 15 | 0.00424638 | PVX_090240(CyRPA) |
| Brazil | 7 | 1115001 | 1116000 | 12 | 0.00375362 | PVX_099915(RNA-binding |
| Brazil | 7 | 1159001 | 1160000 | 14 | 0.0046087 | PVX_099980(MSP1) |
| Brazil | 7 | 1160001 | 1161000 | 23 | 0.00665217 | PVX_099980(MSP1) |
| Brazil | 8 | 1639001 | 1640000 | 14 | 0.00353623 | - |
| Brazil | 8 | 11001 | 12000 | 12 | 0.00356522 | PVX_094230(Hypothetical) |
| Brazil | 9 | 21001 | 22000 | 10 | 0.00346377 | PVX_090840(Hypothetical) |
| Brazil | 9 | 36001 | 37000 | 10 | 0.00371014 | PVX_090860(CPW-WPC) |
| Brazil | 9 | 28001 | 29000 | 15 | 0.00382609 | PVX_090848(tRNA leucine) |
| Brazil | 10 | 1191001 | 1192000 | 12 | 0.00337681 | PVX_97755(Myosin B) |
| Brazil | 10 | 1266001 | 1267000 | 12 | 0.00347826 | - |
| Brazil | 10 | 68001 | 69000 | 9 | 0.00382609 | PVX_079750(Hypothetical) |
| Brazil | 10 | 71001 | 72000 | 14 | 0.00385507 | PVX_079755(phosphoenolpyruva |
| Brazil | 10 | 1270001 | 1271000 | 14 | 0.00389855 | PVX_097665(Hypothetical) |
| Brazil | 10 | 1283001 | 1284000 | 13 | 0.00389855 | PVX_097635(Hypothetical) |
| Brazil | 10 | 1218001 | 1219000 | 16 | 0.00391304 | PVX_097720(MSP3) |
| Brazil | 10 | 1256001 | 1257000 | 17 | 0.00395652 | PVX_097680(MSP3) |
| Brazil | 10 | 1217001 | 1218000 | 10 | 0.00430435 | PVX_097725(MSP3) |
| Brazil | 10 | 1215001 | 1216000 | 15 | 0.00431884 | - |
| Brazil | 10 | 1306001 | 1307000 | 15 | 0.00462319 | PVX_097600(Hypothetical) |
| Brazil | 10 | 1265001 | 1266000 | 15 | 0.00504348 | - |
| Brazil | 10 | 67001 | 68000 | 15 | 0.00518841 | PVX_079750(Hypothetical) |
| Brazil | 12 | 775001 | 776000 | 14 | 0.00378261 | PVX_082655(MSP7) |
| Brazil | 12 | 762001 | 763000 | 13 | 0.00443478 | PVX_082685(MSP7) |
| Brazil | 12 | 766001 | 767000 | 17 | 0.00475362 | PVX_082675(MSP7) |
| Brazil | 12 | 764001 | 765000 | 19 | 0.0054058 | PVX_082680(MSP7) |
| Brazil | 12 | 751001 | 752000 | 16 | 0.0066087 | PVX_082710(Hypothetical) |
| Brazil | 13 | 1999001 | 2000000 | 9 | 0.00331884 | PVX_086315(RPN6) |
| Brazil | 13 | 909001 | 910000 | 11 | 0.00334783 | PVX_085105(TFIID-like) |
| Brazil | 13 | 40001 | 41000 | 11 | 0.00347826 | PVX_084160(dynein heavy chain) |
| Brazil | 13 | 130001 | 131000 | 11 | 0.00410145 | PVX_084260(Hypothetical) |
| Brazil | 13 | 1992001 | 1993000 | 15 | 0.00437681 | PVX_086305(ubiquitin-40S |
| Brazil | 14 | 2032001 | 2033000 | 14 | 0.00382609 | PVX_124075(Hypothetical) |
| Colombia | 1 | 805001 | 806000 | 30 | 0.00639873 | PVX_093715(VIR) |
| Colombia | 2 | 82001 | 83000 | 29 | 0.00600106 | PVX_096980(VIR) |
| Colombia | 2 | 92001 | 93000 | 26 | 0.00620624 | - |
| Colombia | 2 | 2001 | 3000 | 44 | 0.00627393 | PVX_096910(VIR) |
| Colombia | 3 | 22001 | 23000 | 23 | 0.00537282 | PVX_001080(Hypothetical) |
| Colombia | 3 | 902001 | 903000 | 28 | 0.00540455 | - |
| Colombia | 3 | 14001 | 15000 | 19 | 0.0054532 | PVX_001090(Hypothetical) |
| Colombia | 3 | 971001 | 972000 | 25 | 0.00563088 | - |
| Colombia | 3 | 19001 | 20000 | 23 | 0.0057028 | PVX_001080(Hypothetical) |
| Colombia | 3 | 901001 | 902000 | 31 | 0.0060074 | PVX_096001(VIR) |
| Colombia | 3 | 21001 | 22000 | 29 | 0.00607509 | PVX_001080(Hypothetical) |
| Colombia | 3 | 3001 | 4000 | 31 | 0.00609625 | - |

| | | | | | | |
|---|---|---|---|---|---|---|
| Colombia | 3 | 960001 | 961000 | 32 | 0.00654469 | PVX_000010(Plasmodium |
| Colombia | 4 | 51001 | 52000 | 24 | 0.00541089 | - |
| Colombia | 4 | 585001 | 586000 | 26 | 0.00599894 | PVX_003820(SERA) |
| Colombia | 4 | 564001 | 565000 | 39 | 0.00685775 | PVX_003840(SERA) |
| Colombia | 4 | 568001 | 569000 | 31 | 0.00727657 | PVX_003835(SERA) |
| Colombia | 5 | 1358001 | 1359000 | 20 | 0.00547435 | PVX_090330(PvRBP-2) |
| Colombia | 5 | 1291001 | 1292000 | 26 | 0.00643892 | - |
| Colombia | 5 | 1313001 | 1314000 | 32 | 0.00813961 | - |
| Colombia | 6 | 26001 | 27000 | 27 | 0.0057028 | PVX_001645(VIR) |
| Colombia | 6 | 30001 | 31000 | 22 | 0.00610894 | - |
| Colombia | 6 | 24001 | 25000 | 28 | 0.00689159 | PVX_001649(VIR) |
| Colombia | 6 | 25001 | 26000 | 32 | 0.00736542 | - |
| Colombia | 6 | 18001 | 19000 | 27 | 0.00805923 | PVX_001635(VIR) |
| Colombia | 7 | 1471001 | 1472000 | 27 | 0.00563934 | PVX_005045(VIR) |
| Colombia | 7 | 1450001 | 1451000 | 35 | 0.00579164 | PVX_086860(VIR) |
| Colombia | 7 | 1438001 | 1439000 | 39 | 0.00654469 | PVX_086865(VIR) |
| Colombia | 7 | 1451001 | 1452000 | 35 | 0.00696774 | - |
| Colombia | 8 | 7001 | 8000 | 40 | 0.00561396 | PVX_094230(Hypothetical) |
| Colombia | 8 | 24001 | 25000 | 32 | 0.00614278 | PVX_094240(VIR) |
| Colombia | 8 | 1001 | 2000 | 33 | 0.00715812 | - |
| Colombia | 9 | 7001 | 8000 | 32 | 0.00588472 | PVX_090835(VIR) |
| Colombia | 9 | 3001 | 4000 | 33 | 0.00590164 | - |
| Colombia | 9 | 8001 | 9000 | 31 | 0.0066166 | - |
| Colombia | 9 | 9001 | 10000 | 35 | 0.00750502 | - |
| Colombia | 9 | 6001 | 7000 | 37 | 0.00825383 | - |
| Colombia | 10 | 1377001 | 1378000 | 30 | 0.00528821 | - |
| Colombia | 10 | 1223001 | 1224000 | 25 | 0.00549974 | PVX_097715(Hypothetical) |
| Colombia | 10 | 21001 | 22000 | 26 | 0.00564357 | PVX_079695(Hypothetical) |
| Colombia | 10 | 1265001 | 1266000 | 31 | 0.00571973 | - |
| Colombia | 10 | 71001 | 72000 | 25 | 0.0057578 | PVX_079755(phosphoenolpyruva |
| Colombia | 10 | 1253001 | 1254000 | 45 | 0.00602221 | PVX_097680(MSP3) |
| Colombia | 10 | 1256001 | 1257000 | 45 | 0.00624008 | PVX_097680(MSP3) |
| Colombia | 11 | 2062001 | 2063000 | 20 | 0.00533051 | - |
| Colombia | 12 | 2960001 | 2961000 | 18 | 0.00543628 | - |
| Colombia | 13 | 1001 | 2000 | 27 | 0.00530513 | - |
| Colombia | 13 | 2025001 | 2026000 | 17 | 0.00601163 | - |
| Colombia | 13 | 1993001 | 1994000 | 31 | 0.00795769 | PVX_086305(ubiquitin-40S |
| Colombia | 14 | 43001 | 44000 | 25 | 0.00610048 | PVX_121885(CLAG) |
| Mexico | 1 | 829001 | 830000 | 17 | 0.00555903 | PVX_093735(VIR) |
| Mexico | 1 | 805001 | 806000 | 24 | 0.00761309 | PVX_093715(VIR) |
| Mexico | 2 | 1001 | 2000 | 16 | 0.00538265 | PVX_096910(VIR) |
| Mexico | 2 | 92001 | 93000 | 18 | 0.00553058 | PVX_096987(VIR) |
| Mexico | 2 | 2001 | 3000 | 27 | 0.00606543 | PVX_096910(VIR) |
| Mexico | 3 | 923001 | 924000 | 21 | 0.00535989 | PVX_095990(VIR) |
| Mexico | 3 | 19001 | 20000 | 17 | 0.00568421 | PVX_001080(Hypothetical) |
| Mexico | 3 | 959001 | 960000 | 21 | 0.00583784 | - |
| Mexico | 3 | 22001 | 23000 | 16 | 0.00604267 | PVX_001080(Hypothetical) |
| Mexico | 3 | 901001 | 902000 | 29 | 0.00671408 | PVX_096001(VIR) |
| Mexico | 3 | 26001 | 27000 | 19 | 0.00836415 | PVX_001080(Hypothetical) |
| Mexico | 3 | 21001 | 22000 | 25 | 0.00936558 | PVX_001080(Hypothetical) |
| Mexico | 4 | 562001 | 563000 | 20 | 0.00622475 | PVX_003840(SERA) |
| Mexico | 4 | 68001 | 69000 | 25 | 0.00654339 | - |
| Mexico | 4 | 569001 | 570000 | 18 | 0.00656615 | PVX_003835(SERA) |
| Mexico | 4 | 593001 | 594000 | 22 | 0.0065889 | PVX_003810(SERA) |
| Mexico | 5 | 1291001 | 1292000 | 21 | 0.00538265 | - |
| Mexico | 5 | 38001 | 39000 | 18 | 0.00554196 | PVX_088805(VIR) |
| Mexico | 5 | 1358001 | 1359000 | 18 | 0.00559886 | PVX_090330(PvRBP-2) |
| Mexico | 5 | 17001 | 18000 | 21 | 0.00623613 | PVX_088795(VIR) |
| Mexico | 5 | 1256001 | 1257000 | 21 | 0.00706686 | PVX_090240(CyRPA) |
| Mexico | 5 | 1313001 | 1314000 | 27 | 0.0090697 | - |
| Mexico | 6 | 24001 | 25000 | 23 | 0.00637269 | PVX_001640(VIR) |
| Mexico | 6 | 25001 | 26000 | 24 | 0.00700996 | - |
| Mexico | 6 | 18001 | 19000 | 20 | 0.00743101 | PVX_001635(VIR) |
| Mexico | 7 | 1450001 | 1451000 | 25 | 0.00535989 | PVX_086860(VIR) |
| Mexico | 7 | 1451001 | 1452000 | 25 | 0.0058037 | - |
| Mexico | 7 | 970001 | 971000 | 17 | 0.00614509 | PVX_099690(TLP2) |
| Mexico | 7 | 1430001 | 1431000 | 20 | 0.00622475 | PVX_086875(VIR) |

| | | | | | | |
|---|---|---|---|---|---|---|
| Mexico | 7 | 1438001 | 1439000 | 34 | 0.00971835 | PVX_086865(VIR) |
| Mexico | 8 | 1672001 | 1673000 | 16 | 0.0055192 | PVX_119210(VIR) |
| Mexico | 8 | 7001 | 8000 | 26 | 0.00561024 | PVX_094230(Hypothetical) |
| Mexico | 8 | 24001 | 25000 | 21 | 0.00573542 | PVX_094240(VIR) |
| Mexico | 8 | 1001 | 2000 | 28 | 0.00655477 | - |
| Mexico | 9 | 337001 | 338000 | 16 | 0.00579801 | PVX_091200(Hypothetical) |
| Mexico | 10 | 1265001 | 1266000 | 20 | 0.00562162 | - |
| Mexico | 10 | 1221001 | 1222000 | 23 | 0.00629303 | PVX_097720(MSP3) |
| Mexico | 10 | 1218001 | 1219000 | 25 | 0.00674822 | PVX_097720(MSP3) |
| Mexico | 10 | 1226001 | 1227000 | 28 | 0.00685064 | PVX_097710(MSP3) |
| Mexico | 10 | 1256001 | 1257000 | 27 | 0.00784068 | PVX_097680(MSP3) |
| Mexico | 10 | 1220001 | 1221000 | 35 | 0.0093542 | PVX_097720(MSP3) |
| Mexico | 10 | 1219001 | 1220000 | 31 | 0.00983215 | PVX_097720(MSP3) |
| Mexico | 11 | 2027001 | 2028000 | 21 | 0.00594026 | PVX_124725(VIR) |
| Mexico | 11 | 2028001 | 2029000 | 23 | 0.00636131 | - |
| Mexico | 11 | 2062001 | 2063000 | 15 | 0.00714651 | - |
| Mexico | 13 | 5001 | 6000 | 19 | 0.00570128 | - |
| Mexico | 13 | 1993001 | 1994000 | 19 | 0.00614509 | PVX_086305(ubiquitin-40S |
| Mexico | 13 | 6001 | 7000 | 17 | 0.00627027 | PVX_084090(liver stage |
| Mexico | 13 | 2025001 | 2026000 | 22 | 0.00667994 | - |
| Mexico | 14 | 43001 | 44000 | 20 | 0.00629303 | PVX_121885(CLAG) |
| Peru | 2 | 92001 | 93000 | 18 | 0.00544928 | PVX_096987(VIR) |
| Peru | 2 | 66001 | 67000 | 19 | 0.00588213 | PVX_096965(VIR) |
| Peru | 3 | 26001 | 27000 | 18 | 0.00405797 | PVX_001080(Hypothetical) |
| Peru | 3 | 19001 | 20000 | 16 | 0.00428986 | PVX_001080(Hypothetical) |
| Peru | 3 | 22001 | 23000 | 22 | 0.00588213 | PVX_001080(Hypothetical) |
| Peru | 3 | 21001 | 22000 | 27 | 0.00678647 | PVX_001080(Hypothetical) |
| Peru | 4 | 861001 | 862000 | 18 | 0.00411981 | PVX_003485(VIR) |
| Peru | 4 | 872001 | 873000 | 25 | 0.00425121 | PVX_003475(Hypothetical) |
| Peru | 4 | 599001 | 600000 | 18 | 0.00442899 | PVX_003805(SERA) |
| Peru | 4 | 564001 | 565000 | 25 | 0.00465314 | PVX_003840(SERA) |
| Peru | 4 | 569001 | 570000 | 16 | 0.00469952 | PVX_003835(SERA) |
| Peru | 4 | 573001 | 574000 | 18 | 0.00511691 | PVX_003830(SERA) |
| Peru | 4 | 584001 | 585000 | 29 | 0.00524058 | PVX_003820(SERA) |
| Peru | 4 | 175001 | 176000 | 25 | 0.00564251 | - |
| Peru | 4 | 566001 | 567000 | 34 | 0.00578164 | PVX_003840(SERA) |
| Peru | 4 | 585001 | 586000 | 28 | 0.00602899 | PVX_003820(SERA) |
| Peru | 4 | 562001 | 563000 | 32 | 0.00745894 | PVX_003840(SERA) |
| Peru | 5 | 37001 | 38000 | 19 | 0.00419324 | PVX_088805(VIR) |
| Peru | 5 | 1297001 | 1298000 | 16 | 0.00471498 | PVX_090285(Pvstp1) |
| Peru | 6 | 10001 | 11000 | 18 | 0.00405024 | PVX_001625(VIR) |
| Peru | 6 | 18001 | 19000 | 21 | 0.00437488 | PVX_001635(VIR) |
| Peru | 6 | 841001 | 842000 | 26 | 0.0045372 | PVX_110935(Hypothetical) |
| Peru | 6 | 25001 | 26000 | 26 | 0.0051942 | - |
| Peru | 7 | 1401001 | 1402000 | 18 | 0.00409275 | PVX_086900(Plasmodium |
| Peru | 7 | 969001 | 970000 | 22 | 0.00442899 | PVX_099690(TLP2) |
| Peru | 7 | 1356001 | 1357000 | 35 | 0.00462995 | PVX_086940(Hypothetical) |
| Peru | 7 | 970001 | 971000 | 12 | 0.00467633 | PVX_099690(TLP2) |
| Peru | 7 | 1160001 | 1161000 | 25 | 0.00482319 | PVX_099980(MSP1) |
| Peru | 7 | 1116001 | 1117000 | 25 | 0.00511691 | PVX_099915(RNA-binding |
| Peru | 7 | 1044001 | 1045000 | 30 | 0.00523285 | - |
| Peru | 8 | 17001 | 18000 | 16 | 0.00405797 | - |
| Peru | 8 | 8001 | 9000 | 26 | 0.0043285 | PVX_094230(Hypothetical) |
| Peru | 8 | 1001 | 2000 | 29 | 0.005457 | - |
| Peru | 8 | 7001 | 8000 | 29 | 0.00584348 | PVX_094230(Hypothetical) |
| Peru | 9 | 8001 | 9000 | 24 | 0.00428986 | - |
| Peru | 9 | 36001 | 37000 | 17 | 0.00480773 | PVX_090860(CPW-WPC) |
| Peru | 9 | 9001 | 10000 | 33 | 0.00684831 | - |
| Peru | 10 | 1253001 | 1254000 | 23 | 0.00422029 | PVX_097680(MSP3) |
| Peru | 10 | 1248001 | 1249000 | 21 | 0.00449855 | PVX_097685(MSP3) |
| Peru | 10 | 1218001 | 1219000 | 24 | 0.00465314 | PVX_097720(MSP3) |
| Peru | 10 | 1220001 | 1221000 | 35 | 0.00487729 | PVX_097720(MSP3) |
| Peru | 10 | 1223001 | 1224000 | 22 | 0.00544155 | PVX_097715(Hypothetical) |
| Peru | 10 | 1307001 | 1308000 | 18 | 0.00614493 | PVX_097600(Hypothetical) |
| Peru | 12 | 881001 | 882000 | 27 | 0.00403478 | PVX_082480(Hypothetical) |
| Peru | 12 | 2967001 | 2968000 | 16 | 0.0050087 | PVX_118675(Hypothetical) |
| Peru | 12 | 764001 | 765000 | 28 | 0.0064 | PVX_082680(MSP7) |

| | | | | | | |
|---|---|---|---|---|---|---|
| Peru | 13 | 1993001 | 1994000 | 20 | 0.00541063 | PVX_086305(ubiquitin-40S |
| Peru | 14 | 996001 | 997000 | 13 | 0.00431304 | PVX_122920(Hypothetical) |
| Peru | 14 | 2032001 | 2033000 | 17 | 0.00433623 | PVX_124075(Hypothetical) |
| Peru | 14 | 2019001 | 2020000 | 26 | 0.00436715 | PVX_124065(Hypothetical) |
