# Supplementary material for: Genome-wide diversity and differentiation in New World populations of the human malaria parasite Plasmodium vivax
Source: PLoS Negl Trop Dis. 2017 Jul 31;11(7):e0005824. doi: 10.1371/journal.pntd.0005824 (PMC5552344; doi:10.1371/journal.pntd.0005824)
Supplement: S3 Table — (PDF) [file pntd.0005824.s009.pdf]

S3 Table. List of annotated genes mapping to the 50 windows (1 kb-wide) with the lowest Tajima's D values in each New World P. vivax population.

Country	Chromosome	Start (bp)	SNPs	Tajima's D	Gene
Brazil	1	544000	7	-1.1801	
Brazil	1	802000	12	-1.29209	PVX_093710(VIR)
Brazil	2	35000	6	-1.1428	-
Brazil	2	78000	6	-1.1428	-
Brazil	2	266000	6	-1.1428	PVX_081345(secreted ookinete protein. putative (PSOP24))
Brazil	2	501000	5	-1.09607	PVX_081625(hypothetical)
Brazil	3	3000	6	-1.1428	-
Brazil	3	768000	7	-1.1428	PVX_096110(hypothetical)
Brazil	3	896000	6	-1.1428	PVX_096003(VIR)
Brazil	4	68000	7	-1.1801	-
Brazil	4	590000	7	-1.1801	-
Brazil	4	681000	6	-1.1428	PVX_003695(hypothetical)
Brazil	4	872000	7	-1.1801	PVX_003475(hypothetical)
Brazil	5	471000	5	-1.09607	PVX_003950(transport protein SEC61 gamma subunit)
Brazil	5	1284000	5	-1.09607	PVX_090270(tryptophan-rich antigen (Pv-fam-a))
Brazil	5	1296000	11	-1.27605	-
Brazil	5	1299000	5	-1.09607	PVX_090285(Pvstp1)
Brazil	6	18000	7	-1.1801	PVX_001635(VIR)
Brazil	6	20000	5	-1.09607	PVX_001635(VIR)
Brazil	7	537000	5	-1.09607	-
Brazil	7	1265000	6	-1.1428	PVX_087065(hypothetical)
Brazil	7	1378000	8	-1.09607	-
Brazil	7	1424000	6	-1.1428	-
Brazil	8	366000	5	-1.09607	PVX_094595(histone deacetylase 2(HDA2))
Brazil	8	452000	7	-1.1801	PVX_094690(hypothetical)
Brazil	8	625000	5	-1.09607	PVX_094900(hypothetical)
Brazil	8	881000	5	-1.09607	PVX_095195(ATP-dependent RNA helicase DDX6 (DOZI))
Brazil	8	1233000	6	-1.1428	PVX_119735(transporter)
Brazil	9	6000	5	-1.09607	PVX_090835(hypothetical)
Brazil	9	61000	7	-1.1801	PVX_090878(hypothetical). PVX_090880(phenylalanine--tRNA ligase beta subunit)
Brazil	9	452000	5	-1.09607	PVX_091355(hypothetical)
Brazil	10	832000	5	-1.09607	PVX_080660(RNA pseudouridylate synthase)
Brazil	10	854000	6	-1.1428	PVX_080660(RNA pseudouridylate synthase)
Brazil	10	1033000	5	-1.09607	PVX_097940(cell differentiation protein rcd1)
Brazil	10	1054000	7	-1.1801	PVX_097910(hypothetical)
Brazil	10	1220000	5	-1.09607	PVX_097720(MPS3)
Brazil	10	1251000	10	-1.25756	PVX_097685(MSP3)
Brazil	10	1275000	5	-1.09607	PVX_097655(50S ribosomal protein L28. apicoplast)
Brazil	11	396000	5	-1.09607	PVX_115015(protein transport protein Sec24A (SEC24A))
Brazil	11	975000	6	-1.1428	-
Brazil	11	1120000	7	-1.1801	-
Brazil	11	1736000	6	-1.09607	PVX_1153520(MYND finger protein)

Brazil	12	1038000	6	-1.1428	PVX_116515(hypothetical)
Brazil	12	2960000	5	-1.09607	-
Brazil	13	19000	6	-1.1428	PVX_084110(hypothetical)
Brazil	13	865000	6	-1.1428	PVX_085045(WRN)
Brazil	14	16000	6	-1.1428	-
Brazil	14	996000	7	-1.1801	PVX_122920(hypothetical)
Brazil	14	1565000	6	-1.1428	PVX_123600(hypothetical)
Peru	1	372000	10	-1.63117	PVX_088025 (hypothetical)
Peru	1	376000	8	-1.73682	PVX_088025(hypothetical)
Peru	2	264000	13	-1.66682	PVX_081345(secreted ookinete protein (PSOP24))
Peru	3	393000	6	-1.62207	PVX_000660(hypothetical)
Peru	4	571000	10	-1.71995	PVX_003835(SERA)
Peru	4	838000	6	-1.62207	PVX_003510(VIR)
Peru	5	313000	10	-1.63117	PVX_089110(hypothetical)
Peru	5	375000	6	-1.62207	PVX_089200(histone acetyltransferase GCN5)
Peru	5	437000	7	-1.68474	-
Peru	5	1302000	10	-1.81858	-
Peru	5	1303000	8	-1.73682	-
Peru	5	1304000	7	-1.68474	-
Peru	5	1318000	11	-1.66874	-
Peru	6	3000	7	-1.68474	-
Peru	6	32000	15	-1.66628	-
Peru	6	255000	9	-1.78085	PVX_001925(hypothetical)
Peru	6	532000	17	-1.66643	PVX_111365(U3 small nucleolar ribonucleoprotein protein MPP10)
Peru	6	896000	7	-1.68474	-
Peru	7	1468000	6	-1.62207	-
Peru	7	1471000	9	-1.67352	PVX_005045(VIR)
Peru	8	1651000	7	-1.68474	PVX_119225(hypothetical)
Peru	8	1654000	9	-1.67352	-
Peru	9	11000	16	-1.63963	-
Peru	9	32000	14	-1.70377	PVX_090850(FeS cluster assembly protein SufD (SufD))
Peru	9	260000	9	-1.67352	-
Peru	9	1563000	7	-1.68474	PVX_092605(hypothetical)
Peru	9	1788000	6	-1.62207	PVX_092875(dynamin-like protein)
Peru	10	227000	9	-1.78085	PVX_079935(ADP-ribosylation factor GTPase-activating protein (ARF-GAP))
Peru	10	650000	9	-1.67352	PVX_080410(transcription factor with AP2 domain(s)(ApiAP2))
Peru	10	991000	6	-1.62207	PVX_097995(ATP-dependent RNA helicase)
Peru	10	1092000	7	-1.68474	PVX_097850(small ubiquitin-related modifier (SUMO))
Peru	10	1236000	6	-1.62207	PVX_097700(MSP3)
Peru	10	1251000	7	-1.68474	PVX_097685(MSP3)
Peru	10	1256000	35	-1.71586	PVX_097680(MSP3)
Peru	10	1378000	6	-1.62207	PVX_097555(VIR)
Peru	12	974000	6	-1.62207	PVX_082375(PST-A protein)
Peru	12	2642000	7	-1.68474	PVX_118360(TRAP-like protein (TREP))

Peru	12	2727000	7	-1.68474	PVX_118455(clathrin coat assembly protein AP50)
Peru	12	2927000	8	-1.73682	PVX_118648(hypothetical)
Peru	13	790000	12	-1795	PVX_084935(hypothetical). PVX_084940(voltage-dependent anion-selective channel protein). PVX_084945(syntaxin)
Peru	13	799000	9	-1.67352	PVX_084945(syntaxin)
Peru	13	827000	12	-1.62506	PVX_084985(hypothetical)
Peru	13	897000	7	-1.68474	PVX_085045(ADP-dependent DNA helicase RecQ (WRN))
Peru	13	1101000	7	-1.68474	PVX_085320(cyclophilin)
Peru	13	2024000	9	-1.67352	PVX_086345(hypothetical)
Peru	14	43000	14	-1.63655	PVX_121885(cytoadherence linked asexual protein (CLAG))
Peru	14	2062000	8	-1.73682	PVX_124100(T-complex protein 1 subunit gamma (CCT3)). PVX_124105(hypothetical)
Peru	14	2396000	8	-1.73682	PVX_100810(hypothetical)
Peru	14	2803000	11	-1.66874	PVX_101345(hypothetical)
Brazil/Colombia	12	2444000	6/9	-1.1428/-1.80757	-
Colombia	1	544000	7/9	-1.72433	-
Colombia	1	653000	10	-1.78553	PVX_093575(DNA repair protein RAD54)
Colombia	2	88000	9	-1.89082	PVX_096985(VIR)
Colombia	2	267000	8	-1.75028	PVX_081350(hypothetical)
Colombia	3	12000	9	-1.89082	PVX_001095(hypothetical)
Colombia	3	27000	16	-1.99687	PVX_001080(hypothetical)
Colombia	3	908000	23	-1.78135	PVX_096000(VIR)
Colombia	3	909000	18	-1.87495	PVX_096000(VIR)
Colombia	3	936000	14	-1.74934	PVX_103155(VIR)
Colombia	3	1009000	14	-1.88242	-
Colombia	4	1000	11	-1.89924	PVX_106210(VIR)
Colombia	4	93000	13	-1.90759	PVX_002550(hypothetical)
Colombia	4	629000	10	-1.93326	PVX_003770(MSP5)
Colombia	4	867000	29	-2.04754	-
Colombia	5	1238000	11	-1.82827	PVX_090220(hypothetical)
Colombia	5	1322000	25	-1.7768	-
Colombia	5	1341000	18	-1.76742	-
Colombia	6	87000	10	-1.93326	PVX_001720(DNA polymerase delta catalytic subunit)
Colombia	7	111000	7	-1.7834	PVX_098670(prefoldin subunit)
Colombia	7	410000	9	-1.72433	PVX_098965(DNA repair protein REV1)
Colombia	7	505000	18	-1.7439	PV_099100(hypothetical)
Colombia	7	623000	7	-1.7834	PVX_099250(ubiquitin fusion degradation protein 1 (UFD1))
Colombia	7	1336000	10	-1.85666	PVX_086950(gametocyte development protein 1(GDV1))
Colombia	7	1397000	8	-1.75028	PVX_086903(hypothetical)
Colombia	7	1413000	18	-1.99927	PVX_086895(VIR)
Colombia	7	1461000	16	-1.79247	PVX_086845(VIR)
Colombia	8	199000	9	-1.73027	PVX_094385(hypothetical)
Colombia	9	4000	22	-1.8404	-
Colombia	9	691000	12	-1.80428	PVX_091660(hypothetical)
Colombia	9	1773000	8	-1.75028	PVX_092865(protein kinase domain containing protein)
Colombia	10	938000	10	-1.78553	PVX_098050(phosphatidylinositol 4-kinase (PI4K))

Colombia	10	1074000	10	-1.78006	PVX_097885(hypothetical)
Colombia	10	1212000	27	-1.89525	PVX_097730(hypothetical)
Colombia	10	1227000	18	-1.81446	PVX_097710(MSP3)
Colombia	10	1235000	28	-1.83956	PVX_097700(MSP3)
Colombia	10	1249000	12	-1.80428	PVX_097685(MSP3)
Colombia	10	1258000	22	-1.77833	PVX_097675(MSP3)
Colombia	10	1260000	9	-1.80757	PVX_097675(MSP3)
Colombia	10	1275000	9	-1.89082	PVX_097655(50S ribosomal protein L28. apicoplast)
Colombia	10	1399000	13	-1.9695	-
Colombia	10	1411000	16	-1.8445	PVX_097530(VIR)
Colombia	11	10000	11	-1.89924	-
Colombia	11	174000	8	-1.75028	PVX_115290(signal recognition particle receptor)
Colombia	11	2017000	7	-1.7834	-
Colombia	12	811000	9	-1.89082	-
Colombia	12	1830000	10	-1.93326	PVX_117365(hypothetical)
Colombia	13	745000	8	-1.84149	PVX_084870(chromatin assembly factor 1 P55 subunit)
Colombia	14	2064000	9	-1.80757	PVX_124105(hypothetical)
Colombia/Peru	14	389000	15/12	-2.02517/-1.63355	PVX_122222(hypothetical)
Mexico	1	45000	6	-1.5376	PVX_087700(hypothetical)
Mexico	1	163000	4	-1.37697	PVX_087785(hypothetical)
Mexico	1	445000	5	-1.46687	-
Mexico	2	34000	4	-1.37697	PVX_096938(VIR)
Mexico	2	53000	11	-1.53287	-
Mexico	2	133000	4	-1.37697	-
Mexico	2	405000	4	-1.37697	-
Mexico	2	601000	4	-1.37697	PVX_081710(actin-like protein(ALP3))
Mexico	2	623000	4	-1.37697	PVX_081745(RNA-binding protein)
Mexico	3	17000	9	-1.55679	PVX_001085(hypothetical)
Mexico	3	337000	4	-1.37697	PVX_000745(erythrocyte vesicle protein 1(EVP1))
Mexico	3	342000	4	-1.37697	PVX_000737(tRNA Isoleucine)
Mexico	3	449000	4	-1.37697	PVX_000590(eukaryotic translation initiation factor 3 subunit 2)
Mexico	3	864000	4	-1.37697	-
Mexico	3	883000	4	-1.37697	-
Mexico	3	910000	7	-1.44191	PVX_096000(VIR)
Mexico	4	29000	12	-1.47443	-
Mexico	4	321000	4	-1.37697	PVX_002845(hypothetical)
Mexico	4	575000	5	-1.46687	PVX_003830(SERA)
Mexico	4	603000	6	-1.5376	PVX_003800(SERA)
Mexico	4	875000	5	-1.46687	PVX_003475(hypothetical)
Mexico	5	20000	5	-1.46687	-
Mexico	5	1066000	5	-1.46687	PVX_090045(hypothetical)
Mexico	5	1368000	14	-1.39055	PVX_090335(VIR)
Mexico	6	351000	5	-1.46687	-
Mexico	6	1013000	5	-1.46687	PVX_004525(VIR)

Mexico	7	20000	5	-1.46687	PVX_098585(RBP1a)
Mexico	7	1402000	6	-1.5376	PVX_086900(hypothetical)
Mexico	7	1475000	9	-1.55679	PVX_005050(VIR)
Mexico	7	1476000	6	-1.5376	PVX_005050(VIR)
Mexico	7	1490000	6	-1.5376	-
Mexico	8	19000	5	-1.46687	PVX_094235(hypothetical)
Mexico	8	345000	5	-1.46687	PVX_094580(transcription factor with AP2 domain(s) (ApiAP2))
Mexico	8	1434000	7	-1.5949	PVX_119500(hypothetical)
Mexico	8	1460000	10	-1.60121	PVX_119450(hypothetical). PVX_119445(FAD-dependent glycerol-3-phosphate dehydrogenase)
Mexico	9	58000	6	-1.5376	PVX_090876(CCR4-NOT transcription complex subunit 1)
Mexico	9	101000	5	-1.46687	-
Mexico	9	979000	5	-1.46687	PVX_091950(autophagy-related protein 23 (ATG23))
Mexico	10	22000	8	-1.64233	PVX_079700(hypothetical)
Mexico	10	834000	14	-1.64079	PVX_080660(RNA pseudouridylate synthase)
Mexico	10	1227000	8	-1.50449	PVX_097710(MSP3)
Mexico	11	11000	9	-1.43129	-
Mexico	11	1681000	5	-1.46687	PVX_113590(hypothetical)
Mexico	11	1726000	9	-1.55679	PVX_113535(hypothetical)
Mexico	12	15000	5	-1.46687	-
Mexico	12	774000	10	-1.38512	-
Mexico	12	1086000	6	-1.5376	PVX_116555(hypothetical)
Mexico	12	2817000	7	-1.44191	PVX_118540(copper transporter)
Mexico	14	631000	7	-1.44191	PVX_122500(hypothetical)
Mexico	14	1919000	5	-1.46687	PVX_123945(haloacid dehalogenase-like hydrolase (HAD2))
