# Supplementary material for: Genome-wide diversity and differentiation in New World populations of the human malaria parasite Plasmodium vivax
Source: PLoS Negl Trop Dis. 2017 Jul 31;11(7):e0005824. doi: 10.1371/journal.pntd.0005824 (PMC5552344; doi:10.1371/journal.pntd.0005824)
Supplement: S4 Table — (PDF) [file pntd.0005824.s010.pdf]

S4 Table. List of annotated genes mapping to the 50 windows (1 kb-wide) with the highest Tajima's D values in each New World P. vivax population.

Country	Chromosome	Start (bp)	SNPs	Tajima's D	Genes
Brazil	1	379000	5	2.05589	-
Brazil	2	642000	2	2.02478	PVX_081770(Hypothetical)
Brazil	3	607000	4	2.42111	PVX_096310(Hypothetical)
Brazil	3	656000	2	2.02478	PVX_096265(40S ribosomal protein S5)
Brazil	4	157000	2	2.02478	PVX_002645(Hypothetical)
Brazil	4	518000	3	2.31158	PVX_003900(6-cysteine)
Brazil	4	815000	4	2.06658	-
Brazil	7	36000	2	2.02478	-
Brazil	7	129000	2	2.02478	PVX_098690(copper-transporting ATPase (CuTP))
Brazil	7	348000	2	2.02478	PVX_098910(exoribonuclease)
Brazil	7	438000	6	2.24788	PVX_099005(cysteine repeat modular protein 1. putative (CRMP1))
Brazil	7	657000	2	2.02478	-
Brazil	7	969000	3	2.09389	PVX_099690(thioredoxin-like protein 2 (TLP2))
Brazil	7	1406000	2	2.02478	-
Brazil	8	262000	3	2.31158	-
Brazil	8	538000	2	2.02478	PVX_094810(PRE-binding protein(PREBP))
Brazil	8	683000	2	2.02478	-
Brazil	8	808000	2	2.02478	-
Brazil	8	1068000	2	2.02478	PVX_094450(Hypothetical). PVX_094455(Hypothetical)
Brazil	8	1362000	2	2.02478	PVX_119585(asparagine synthetase [glutamine-hydrolyzing])
Brazil	8	1565000	3	2.31158	PVX_119315(Hypothetical)
Brazil	8	1625000	5	2.43113	PVX_119250(serine/threonine protein kinase (SRPK1))
Brazil	9	26000	6	2.05226	-
Brazil	9	1125000	3	2.09389	-
Brazil	9	1196000	2	2.02478	PVX_092230(Hypothetical)
Brazil	9	1705000	5	2.05589	PVX_0192760(transcription factor with AP2 domain(s) (AP2-O))
Brazil	10	227000	3	2.09389	PVX_079935(ADP-ribosylation factor GTPase-activating protein. putative (ARF-GAP))
Brazil	10	370000	3	2.20274	PVX_080105(Hypothetical)
Brazil	10	1063000	6	2.18268	PVX_097895(TBC domain)
Brazil	10	1217000	10	2.05744	PVX_097725(MSP3)
Brazil	11	434000	2	2.02478	PVX_1149990(guanylyl cyclase)
Brazil	11	1489000	2	2.02478	-
Brazil	11	1920000	2	2.02478	PVX_113315(Hypothetical)
Brazil	12	751000	14	2.26043	PVX_082710(Hypothetical)
Brazil	12	1769000	2	2.02478	PVX_117320(Hypothetical)
Brazil	12	1831000	4	2.42111	PVX_117365(Hypothetical)
Brazil	12	1990000	2	2.02478	PVX_117595(Hypothetical). PVX_117600(Hypothetical). PVX_117605(thioredoxin 1(TRX1))
Brazil	12	2633000	2	2.02478	PVX_118355(Hypothetical)
Brazil	12	2908000	4	2.06658	PVX_118625(trailer hitch homolog (CITH))

Brazil	13	43000	5	2.13094	PVX_084160(dynein heavy chain)
Brazil	13	330000	2	2.02478	PVX_084460(Hypothetical)
Brazil	13	1013000	2	2.02478	PVX_085205(ABC transporter G family member 2 (ABCG2))
Brazil	13	1638000	2	2.02478	-
Brazil	14	265000	3	2.09389	PVX_122077(NLI interacting factor-like phosphatase(NIF1))
Brazil	14	388000	2	2.02478	PVX_122222(Hypothetical)
Brazil	14	669000	3	2.20274	PVX_122530(telomerase reverse transcriptase (TERT))
Brazil	14	2786000	4	2.06658	-
Brazil	14	2974000	4	2.15521	PVX_101505
Peru	1	45000	7	2.03562	PVX_087700(Hypothetical)
Peru	1	546000	2	1.92179	-
Peru	1	778000	4	2.22301	-
Peru	2	680000	3	2.04423	-
Peru	3	114000	3	1.90109	PVX_001010(Hypothetical)
Peru	3	160000	2	2.04552	PVX_000970(pre-mRNA-processing-splicing factor 8)
Peru	4	753000	8	2.21966	PVX_003590(serine/threonine-specific protein kinase)
Peru	5	496000	2	1.89085	PVX_089365(helicase)
Peru	5	847000	2	2.20019	PVX_089810(RAD protein (Pv-fam-e))
Peru	5	952000	4	2.26227	PVX_089935(Hypothetical)
Peru	7	131000	3	2.16352	PVX_098690(copper-transporting ATPase (CuTP))
Peru	7	628000	2	2.13832	PVX_099255(methyltransferase). PVX_099257(RNA-binding protein musash (HoMu))
Peru	7	928000	2	2.13832	-
Peru	7	970000	12	2.1392	PVX_099690(thioredoxin-like protein 2 (TLP2))
Peru	7	1286000	3	2.16352	PVX_087040(gamma-tubulin complex component)
Peru	8	123000	3	2.09195	-
Peru	8	214000	4	2.30153	PVX_094400(60S ribosomal protein L13a)
Peru	8	232000	2	2.13832	PVX_094410(E3 ubiquitin-protein ligase)
Peru	8	301000	2	1.92179	PVX_094510(Hypothetical)
Peru	8	1427000	3	2.33051	PVX_119505(Hypothetical)
Peru	8	1538000	5	2.22595	PVX_119355(circumsporozoite (CS) protein (CSP))
Peru	9	187000	2	2.26206	-
Peru	9	791000	4	2.32116	PVX_091755(calcium-dependent protein kinase 6 (CDPK6))
Peru	9	968000	5	2.39355	-
Peru	10	352000	2	2.07646	-
Peru	10	363000	2	1.98366	PVX_080100(multidrug resistance protein 1. putative (MDR1))
Peru	10	1033000	7	2.54472	PVX_097940(cell differentiation protein rcd1)
Peru	10	1090000	2	2.04552	PVX_097852(conserved Plasmodium protein. unknown function)
Peru	11	48000	2	1.89085	-
Peru	11	724000	3	1.90109	PVX_114670(ER lumen protein retaining receptor (ERD2))
Peru	11	1205000	2	2.13832	PVX_114167(tRNA Leucine). PVX_114165(Hypothetical)
Peru	11	1486000	3	2.35437	PVX_113815(Hypothetical)

Peru	11	1887000	3	2.25894	PVX_113355(centrosomal protein CEP76 (CEP76))
Peru	12	156000	2	2.23113	PVX_083385(Hypothetical)
Peru	12	279000	3	1.99652	-
Peru	12	1092000	2	2.07646	PVX_116560(RNA-binding protein)
Peru	12	1223000	3	2.09195	-
Peru	12	1513000	4	2.24264	PVX_117060(Hypothetical)
Peru	12	1686000	3	2.16352	PVX_117225(ATP synthase (C/AC39) subunit)
Peru	12	2255000	3	2.23509	-
Peru	12	2626000	4	2.20338	PVX_118345(protein transport protein SEC7(SEC7))
Peru	14	725000	6	2.05835	PVX_122620(exportin-1)
Peru	14	1371000	2	1.92179	PVX_123340(eukaryotic translation initiation factor 3 subunit 10)
Peru	14	1514000	3	1.94881	-
Colombia	2	297000	7	2.39878	PVX_081390(Hypothetical)
Colombia	3	23000	7	2.18247	PVX_001080(Hypothetical)
Colombia	3	72000	3	2.40111	PVX_001040(transcription factor with AP2 domain(s)(ApiAP2))
Colombia	3	128000	8	2.79056	PVX_000995(6-cysteine protein (P41))
Colombia	3	622000	3	2.12708	PVX_096295(Hypothetical)
Colombia	4	533000	2	2.1631	PVX_003870(Hypothetical)
Colombia	5	471000	3	2.49246	PVX_089325(2-oxoglutarate dehydrogenase E1 component. mitochondrial precursor)
Colombia	5	742000	4	2.40908	-
Colombia	5	1036000	2	2.07883	-
Colombia	6	155000	2	2.07883	-
Colombia	7	1037000	2	2.07883	PVX_099770(Hypothetical)
Colombia	7	1295000	7	2.70884	PVX_087025(delta tubulin). PVX_087020(Hypothetical)
Colombia	7	1325000	2	2.33165	PVX_086960(vacuolar protein sorting-associated protein 33 (VPS33))
Colombia	8	214000	4	2.63527	PVX_094400(60S ribosomal protein L13a)
Colombia	8	604000	10	2.46045	PVX_094885(Hypothetical)
Colombia	8	618000	2	2.33165	PVX_094895(phospholipase C-like)
Colombia	8	794000	5	2.21599	PVX_095095(Hypothetical)
Colombia	8	1642000	9	2.56281	-
Colombia	9	131000	2	2.19681	PVX_090960(translocon component PTEX88)
Colombia	9	836000	3	2.12708	PVX_091775(leucine-rich repeat protein (LRR11))
Colombia	9	973000	2	2.07883	-
Colombia	9	1101000	2	2.09568	-
Colombia	9	1213000	2	2.1631	PVX_092260(Hypothetical)
Colombia	11	31000	10	2.26894	-
Colombia	11	906000	5	3.10114	PVX_114510(Hypothetical)
Colombia	11	985000	2	2.14625	PVX_114410(oxidoreductase. short-chain dehydrogenase family)
Colombia	11	1151000	2	2.33165	PVX_114240(Hypothetical)
Colombia	11	1513000	4	2.87222	-
Colombia	11	1750000	2	2.12939	PVX_113505(coatomer alpha subunit)

Colombia	12	136000	2	2.33165	-
Colombia	12	577000	6	3.25806	PVX_082935(nucleolar complex protein 2)
Colombia	12	2720000	2	2.07883	PVX_118450(transporter)
Colombia	13	731000	4	2.16136	PVX_084860(Hypothetical)
Colombia	13	1320000	2	2.24738	PVX_085580(rap guanine nucleotide exchange factor (EPAC))
Colombia	13	1774000	3	2.29672	PVX_086035(transcription factor with AP2 domain(s) (AP2-G2))
Colombia	13	1999000	12	2.11611	PVX_086315(26S proteasome regulatory subunit RPN6)
Colombia	14	1304000	2	2.19681	-
Colombia	14	1642000	2	2.09568	PVX_123670(Hypothetical)
Colombia	14	1767000	2	2.17996	-
Colombia	14	2249000	6	2.15049	PVX_100635(Hypothetical)
Mexico	1	235000	6	2.76409	PVX_087865(Hypothetical)
Mexico	1	324000	5	2.4974	PVX_087970(heat shock protein 110 (HSP110c))
Mexico	2	662000	8	2.7341	PVX_081792(Hypothetical)
Mexico	3	9000	8	2.54457	PVX_001100(RAD protein (Pv-fam-e))
Mexico	3	11000	6	2.91465	PVX_001095(Hypothetical)
Mexico	3	20000	7	2.9372	PVX_001080(Hypothetical)
Mexico	3	26000	19	2.81823	PVX_001080(Hypothetical)
Mexico	3	393000	5	2.94061	PVX_000660(Hypothetical)
Mexico	3	615000	6	2.95767	PVX_096300(Hypothetical)
Mexico	3	960000	9	2.93007	-
Mexico	3	962000	5	2.47278	PVX_000010(Hypothetical)
Mexico	4	46000	7	2.65035	PVX_002505(Hypothetical)
Mexico	4	583000	11	2.43712	PVX_003820(SERA)
Mexico	4	591000	9	2.99282	PVX_003810(SERA)
Mexico	4	757000	5	2.57127	-
Mexico	5	111000	7	2.5165	-
Mexico	5	1236000	6	2.5275	-
Mexico	7	40000	3	2.50787	PVX_098610(Hypothetical)
Mexico	7	1123000	4	2.49458	PVX_099930(high molecular weight rhoptry protein 2 (RhopH2))
Mexico	7	1322000	6	2.67806	PVX_086970(Hypothetical)
Mexico	8	276000	11	2.58366	PVX_094470(Hypothetical)
Mexico	8	965000	4	2.61015	PVX_095290(Hypothetical)
Mexico	8	1119000	5	2.42353	PVX_095452(Hypothetical)
Mexico	8	1643000	8	3.06147	-
Mexico	9	223000	3	2.43748	PVX_019065(transcription factor with AP2 domain(s)(ApiAP2))
Mexico	9	310000	4	2.4079	-
Mexico	9	559000	5	2.62051	PVX_091480(Hypothetical)
Mexico	9	1524000	5	2.79287	PVX_092570(transcription factor with AP2 domain(s)(ApiAP2))
Mexico	9	1907000	3	2.43748	PVX_092985(serine/threonine protein kinase)
Mexico	10	17000	4	2.46569	PVX_079695(Hypothetical)

Mexico	10	33000	5	2.69438	PVX_079710(Hypothetical)
Mexico	10	53000	6	2.44146	PVX_079730(Hypothetical)
Mexico	10	1283000	8	2.42396	PVX_097635(Hypothetical)
Mexico	10	1307000	10	2.53338	PVX_097600(Hypothetical)
Mexico	11	396000	4	2.75461	PVX_115015(protein transport protein Sec24A (SEC24A))
Mexico	11	1543000	4	2.49458	PVX_113731(Hypothetical)
Mexico	11	1765000	3	2.47267	PVX_113495(polypyrimidine tract binding protein)
Mexico	11	2062000	15	3.22327	-
Mexico	12	2967000	8	2.85471	PVX_118675(Hypothetical)
Mexico	13	40000	8	2.99255	PVX_084160(dynein heavy chain)
Mexico	13	1988000	5	2.86674	PVX_086295(U2 snRNP-associated SURP motif-containing protein (SR140))
Mexico	14	1329000	4	2.61015	PVX_12330(Hypothetical)
Mexico	14	1645000	4	2.43679	PVX_123675(Hypothetical)
Mexico	14	2808000	4	2.49458	PVX_101335(26S protease regulatory subunit 8 (RPT6))
Mexico	14	2928000	5	2.59589	PVX_101485(rhoptry neck protein 3 (RON3))
Colombia/Mexico	3	961000	11/11	2.6783/2.75685	-
Colombia/Mexico/Peru	4	540000	4 /4/5	2.65681/2.46569 /2.24271	PVX_003865(Hypothetical)
Colombia/Mexico	6	907000	4/3	2.67835/2.66793	PVX_110855(Hypothetical)
Colombia/Peru	9	46000	7/8	2.41321/1.91351	-
Colombia/Peru	9	588000	2/2	2.28109/1.89085	PVX_091530(Hypothetical)
Brazil/Colombia	11	1261000	2/2	2.02478/2.14625	PVX_114090(Hypothetical)
Colombia/Mexico	12	1223000	3/4	2.67515/2.49458	-
Colombia/Peru	12	2300000	2/2	2.14625/1.89085	PVX_117970(flavodoxin domain containing protein)
Colombia/Peru	12	2698000	2/2	2.19681/2.16926	PVX_118425(serine/threonine protein kinase)
Mexico/Peru	13	1905000	4/5	2.52347/2.05835	-
Brazil/Colombia	14	1465000	3/2	2.14625/2.09389	PVX_123480(serine-tRNA ligase)
