# Supplementary material for: Genome-wide diversity and differentiation in New World populations of the human malaria parasite Plasmodium vivax
Source: PLoS Negl Trop Dis. 2017 Jul 31;11(7):e0005824. doi: 10.1371/journal.pntd.0005824 (PMC5552344; doi:10.1371/journal.pntd.0005824)
Supplement: S5 Table — (PDF) [file pntd.0005824.s011.pdf]

S5 Table. List of SNPs with the 100 highest average Wright's fixation index FST values in pairwise comparisons of New World P. vivax populations.

Chromossome	Position	Gene	BRA vs. PER	BRA vs. MEX	BRA vs. COL	PER vs. COL	PER vs. MEX	COL vs. MEX	Average FST
1	28981	Intergenic	0.000000	0.501616	0.343183	0.441713	0.727273	0.000000	0.335631
1	136250	Intergenic	0.631753	0.756326	0.824014	0.031198	0.000000	0.000000	0.373882
1	232875	PVX_087865 (Hypothetical)	0.543565	0.616575	0.835256	0.108020	0.000000	0.055979	0.359899
3	26623	PVX_001080(Hypothetical)	0.000000	0.545436	0.000000	0.000000	0.818182	0.656407	0.336671
3	361385	PVX_000710(Hypothetical)	0.864110	0.690196	0.496965	0.151004	0.000000	0.004752	0.367838
3	438741	PVX_000606(transcription initiation factor IIA subunit 1)	0.506719	0.000000	0.209968	0.059418	0.909091	0.312647	0.332974
3	472863	Intergenic	0.506719	0.345905	0.576981	0.000000	0.909091	0.003932	0.390438
3	501535	PVX_000530(Hypothetical)	0.610833	0.662679	0.750362	0.000000	0.000000	0.000000	0.337312
3	615889	PVX_096300(Hypothetical)	0.528867	0.107866	0.678047	0.000000	0.727273	0.299097	0.390192
3	748831	Intergenic	0.560827	0.756326	0.824014	0.077707	0.000000	0.000000	0.369812
3	831327	Intergenic	0.698488	0.662679	0.750362	0.000000	0.000000	0.000000	0.351922
4	87300	Intergenic	0.698488	0.662679	0.750362	0.000000	0.000000	0.000000	0.351922
4	421618	PVX_002965(ubiquinol-cytochrome-c reductase complex assembly factor 1)	0.784309	0.756326	0.824014	0.000000	0.000000	0.000000	0.394108
4	562234	Intergenic	0.400022	0.000000	0.290413	0.000000	0.909091	0.479222	0.346458

4	581833	PVX_003825(SERA)	0.864110	0.766375	0.413779	0.215759	0.000000	0.111729	0.395292
4	585280	PVX_003820(SERA)	0.493284	0.756326	0.824014	0.126417	0.000000	0.000000	0.366674
4	733723	Intergenic	0.706195	0.756326	0.824014	0.000000	0.000000	0.000000	0.381089
4	743201	PVX_003605(Hypothetical)	0.485967	0.766375	0.835256	0.155404	0.000000	0.000000	0.373834
5	529185	PVX_089400(Hypothetical)	0.698488	0.662679	0.750362	0.000000	0.000000	0.000000	0.351922
5	651962	Intergenic	0.631753	0.582380	0.434099	0.011565	0.727273	0.000000	0.397845
5	935025	PVX_089910(histone acetyltransferase)	0.784309	0.158908	0.221656	0.282662	0.909091	0.000000	0.392771
5	1026978	Intergenic	0.610833	0.167547	0.428548	0.006414	0.818182	0.035508	0.344505
5	1196524	PVX_090155(tubulin alpha chain)	0.560827	0.501616	0.642028	0.000000	0.636364	0.000000	0.390139
5	1265143	Intergenic	0.485967	0.410391	0.835256	0.155404	0.363636	0.236549	0.414534
6	240177	Intergenic	0.560827	0.756326	0.057742	0.263870	0.000000	0.426981	0.344291
6	456217	PVX_111445(Hypothetical)	0.631753	0.756326	0.642028	0.000000	0.000000	0.000000	0.338351
7	124507	Intergenic	0.203501	0.756326	0.760617	0.303592	0.000000	0.000000	0.337339
7	177877	PVX_098712(RhopH3)	0.698488	0.662679	0.750362	0.000000	0.000000	0.000000	0.351922
7	191151	PVX_098725(autophagy-related protein 3)	0.698488	0.233873	0.153332	0.248965	0.909091	0.000000	0.373958
7	670313	PVX_099315(heat shock protein 70)	0.795361	0.766375	0.835256	0.000000	0.000000	0.000000	0.399499

7	735959	Intergenic	0.698488	0.662679	0.750362	0.000000	0.000000	0.000000	0.351922
7	997327	PVX_099725(protein kinase)	0.706195	0.756326	0.700004	0.000000	0.000000	0.000000	0.360421
7	1090586	PVX_099875(Hypothetical)	0.698488	0.662679	0.750362	0.000000	0.000000	0.000000	0.351922
8	178012	PVX_094355(Hypothetical)	0.763993	0.794156	0.950723	0.077707	0.000000	0.061893	0.441412
8	279254	Intergenic	0.665018	0.845199	0.835256	0.021771	0.000000	0.000000	0.394541
8	526326	Intergenic	0.665018	0.845199	0.890487	0.077707	0.000000	0.000000	0.413069
8	543365	Intergenic	0.698488	0.000000	0.428548	0.059418	0.909091	0.312647	0.401365
8	555370	Intergenic	0.018930	0.766375	0.454700	0.267189	0.454545	0.082493	0.340705
8	658857	PVX_094930(Hypothetical)	0.610833	0.385877	0.277477	0.085134	0.818182	0.000000	0.362917
8	956293	Intergenic	0.000000	0.690196	0.298685	0.352600	0.818182	0.138280	0.382991
8	1175938	Intergenic	0.000000	0.616575	0.335807	0.324769	0.727273	0.052414	0.342806
9	26814	Intergenic	0.610833	0.662679	0.750362	0.000000	0.000000	0.000000	0.337312
9	31160	PVX_090850(FeS cluster assembly protein SufD)	0.706195	0.756326	0.300737	0.146199	0.000000	0.187018	0.349413
9	548965	Intergenic	0.000000	0.476721	0.540625	0.465251	0.636364	0.000000	0.353160
9	1120068	Intergenic	0.610833	0.000000	0.375029	0.030364	0.818182	0.203402	0.339635
9	1241520	PVX_092300(Hypothetical)	0.784309	0.756326	0.824014	0.000000	0.000000	0.000000	0.394108

9	1294154	Intergenic	0.000000	0.545436	0.000000	0.000000	0.909091	0.599255	0.342297
9	1465291	PVX_092535(Adenylate and Guanylate cyclase catalytic domain containing protein)	0.610833	0.662679	0.750362	0.000000	0.000000	0.000000	0.337312
9	1616315	PVX_092650(Hypothetical)	0.784309	0.756326	0.387618	0.151004	0.000000	0.124670	0.367321
10	49396	Intergenic	0.631753	0.667249	0.824014	0.031198	0.000000	0.003932	0.359691
10	388625	Intergenic	0.706195	0.283541	0.642028	0.000000	0.818182	0.116252	0.427700
10	441148	Intergenic	0.509222	0.000000	0.586657	0.000000	0.818182	0.361280	0.379224
10	600847	PVX_080335(ApiAP2)	0.706195	0.424892	0.260238	0.178474	0.818182	0.000000	0.397997
10	604999	Intergenic	0.055662	0.766375	0.540625	0.297207	0.363636	0.029215	0.342120
10	657735	PVX_080415(ubiquitin carboxyl-terminal hydrolase 2)	0.631753	0.756326	0.824014	0.031198	0.000000	0.000000	0.373882
10	814705	Intergenic	0.429015	0.756326	0.824014	0.176041	0.000000	0.000000	0.364233
10	1023097	PVX_097955(Hypothetical)	0.452306	0.563729	0.485421	0.000000	0.636364	0.000000	0.356303
10	1292091	Intergenic	0.493284	0.756326	0.760617	0.062957	0.000000	0.000000	0.345531
11	646951	Intergenic	0.430366	0.845199	0.835256	0.204164	0.000000	0.000000	0.385831
11	1277240	PVX_114075(Hypothetical)	0.310000	0.756326	0.824014	0.275752	0.000000	0.000000	0.361015
11	1325302	Intergenic	0.665018	0.115443	0.496965	0.000000	0.636364	0.177734	0.348587
11	1453097	PVX_113844(Hypothetical)	0.784309	0.000000	0.031150	0.494042	0.909091	0.000000	0.369765

12	101922	PVX_083465(60S ribosomal protein L17)	0.631753	0.756326	0.824014	0.031198	0.000000	0.000000	0.373882
12	182650	Intergenic	0.603224	0.225732	0.632349	0.000000	0.545455	0.190347	0.366185
12	182991	Intergenic	0.167184	0.233873	0.234076	0.000000	0.909091	0.663118	0.367890
12	183036	Intergenic	0.603224	0.346431	0.781911	0.018884	0.545455	0.231557	0.421244
12	267594	Intergenic	0.560827	0.582380	0.760617	0.021771	0.636364	0.005119	0.427846
12	355046	PVX_083165(Hypothetical)	0.631753	0.756326	0.760617	0.000000	0.000000	0.000000	0.358116
12	407859	Intergenic	0.310000	0.667249	0.824014	0.275752	0.000000	0.003932	0.346825
12	514569	Intergenic	0.795361	0.346431	0.730365	0.000000	0.000000	0.173378	0.340923
12	556841	Intergenic	0.018930	0.766375	0.632349	0.443417	0.454545	0.000000	0.385936
12	754937	Intergenic	0.610833	0.306670	0.428548	0.006414	0.818182	0.000000	0.361774
12	907576	PVX_082440(Hypothetical)	0.421093	0.067605	0.586657	0.000000	0.727273	0.236549	0.339863
12	1378769	Intergenic	0.000000	0.690196	0.000000	0.000000	0.909091	0.765129	0.394069
12	1452039	Intergenic	0.698488	0.662679	0.678047	0.000000	0.000000	0.000000	0.339869
12	1759482	PVX_117310(Hypothetical)	0.493284	0.756326	0.824014	0.126417	0.000000	0.000000	0.366674
12	2127203	PVX_117770(Hypothetical)	0.452306	0.385877	0.545822	0.000000	0.636364	0.000000	0.336728
12	2265116	PVX_117925(elongation factor 2)	0.560827	0.756326	0.824014	0.077707	0.000000	0.000000	0.369812

12	2479491	Intergenic	0.864110	0.845199	0.890487	0.000000	0.000000	0.000000	0.433299
12	2725612	PVX_118455(clathrin coat assembly protein AP50)	0.729032	0.115443	0.162142	0.324769	0.727273	0.000000	0.343110
12	2736446	Intergenic	0.506719	0.008146	0.026806	0.643177	0.909091	0.000000	0.348990
13	233638	PVX_084370(eukaryotic translation initiation factor 5)	0.429015	0.756326	0.824014	0.176041	0.000000	0.000000	0.364233
13	367258	Intergenic	0.560827	0.667249	0.760617	0.021771	0.000000	0.000000	0.335077
13	555859	Intergenic	0.429015	0.667249	0.824014	0.176041	0.000000	0.003932	0.350042
13	1074238	PVX_085295(Hypothetical)	0.560827	0.582380	0.260238	0.068023	0.636364	0.078470	0.364384
13	1505301	PVX_085765(Hypothetical)	0.429015	0.582380	0.824014	0.176041	0.000000	0.061893	0.345557
13	1585543	Intergenic	0.000000	0.476721	0.496965	0.422701	0.636364	0.000000	0.338792
13	1744515	PVX_086015(Hypothetical)	0.631753	0.219050	0.434099	0.011565	0.727273	0.017576	0.340219
14	246004	PVX_122050(CorA-like Mg2+ transporter protein)	0.631753	0.756326	0.824014	0.031198	0.000000	0.000000	0.373882
14	549324	Intergenic	0.000000	0.476721	0.540625	0.583195	0.818182	0.000000	0.403121
14	593080	PVX_122465(Hypothetical)	0.000000	0.616575	0.162142	0.282662	0.909091	0.209260	0.363288
14	942871	Intergenic	0.493284	0.756326	0.824014	0.126417	0.000000	0.000000	0.366674
14	1451841	PVX_123457(Hypothetical)	0.485967	0.845199	0.890487	0.225936	0.000000	0.000000	0.407932
14	1676804	PVX_123700(Hypothetical)	0.000000	0.476721	0.000000	0.000000	0.909091	0.663118	0.341488

14	1944451	Intergenic	0.430366	0.845199	0.890487	0.275752	0.000000	0.000000	0.406967
14	2012680	Intergenic	0.709062	0.729099	0.724259	0.000000	0.000000	0.000000	0.360403
14	2252614	PVX_100635(Hypothetical)	0.864110	0.845199	0.374161	0.248965	0.000000	0.219323	0.425293
14	2335971	PVX_100730(serine hydroxymethyltransferase)	0.706195	0.103426	0.184979	0.245811	0.818182	0.000000	0.343099
14	2504870	Intergenic	0.430366	0.476721	0.835256	0.204164	0.000000	0.174313	0.353470
14	2887273	PVX_101435(DNA repair protein rhp16)	0.631753	0.352189	0.482695	0.000000	0.727273	0.000000	0.365652
